# Supplementary material for: Can we collect health-related quality of life information from anticoagulated atrial fibrillation participants who have recently experienced a bleed? An observational feasibility study in primary and secondary care in Wales and through a UK online forum
Source: BMJ Open. 2023 Oct 6;13(10):e075335. doi: 10.1136/bmjopen-2023-075335 (PMC10565208; doi:10.1136/bmjopen-2023-075335)
Supplement: Author's manuscript [file bmjopen-2023-075335.draft_revisions.pdf]

BMJ Open

BMJ Open is committed to open peer review. As part of this commitment we make the peer review history of every article we publish publicly available.

When an article is published we post the peer reviewers' comments and the authors' responses online. We also post the versions of the paper that were used during peer review. These are the versions that the peer review comments apply to.

The versions of the paper that follow are the versions that were submitted during the peer review process. They are not the versions of record or the final published versions. They should not be cited or distributed as the published version of this manuscript.

BMJ Open is an open access journal and the full, final, typeset and author-corrected version of record of the manuscript is available on our site with no access controls, subscription charges or pay-per-view fees (http://bmjopen.bmj.com).

If you have any questions on BMJ Open's open peer review process please email info.bmjopen@bmj.com

BMJ Open

Observational Feasibility Study to Investigate the Quality of Life of Anticoagulated Participants with Atrial Fibrillation Following a Bleed

Journal:	*BMJ Open*
Manuscript ID	bmjopen-2023-075335
Article Type:	Original research
Date Submitted by the Author:	05-May-2023

Complete List of Authors:	Hutchings, Hayley; Swansea University, Swansea University Medical School<br>Lanyon, Kirsty; Swansea University, Swansea University Medical School<br>Holland, Gail; Swansea University, Swansea University Medical School<br>Alikhan, Raza; University Hospital of Wales, Department of Haematology<br>Jenkins, Rhys; Swansea City GP Cluster<br>LAING, HAMISH; Swansea University, VBHC Academy, School of Management<br>Hughes, Arfon; Swansea University<br>Lobban, Trudie; Arrythmia Alliance<br>Pollock, Kevin; Bristol-Myers Squibb Pharmaceuticals Ltd<br>Tod, Daniel; Swansea University, Swansea University Medical School<br>Lister, Steven; Bristol-Myers Squibb Pharmaceuticals Ltd

Keywords:	Quality of Life, Feasibility Studies, Bleeding disorders & coagulopathies < HAEMATOLOGY, Anticoagulation < HAEMATOLOGY

SCHOLARONE™
Manuscripts

I, the Submitting Author has the right to grant and does grant on behalf of all authors of the Work (as defined in the below author licence), an exclusive licence and/or a non-exclusive licence for contributions from authors who are: i) UK Crown employees; ii) where BMJ has agreed a CC-BY licence shall apply, and/or iii) in accordance with the terms applicable for US Federal Government officers or employees acting as part of their official duties; on a worldwide, perpetual, irrevocable, royalty-free basis to BMJ Publishing Group Ltd ("BMJ") its licensees and where the relevant Journal is co-owned by BMJ to the co-owners of the Journal, to publish the Work in this journal and any other BMJ products and to exploit all rights, as set out in our licence.

The Submitting Author accepts and understands that any supply made under these terms is made by BMJ to the Submitting Author unless you are acting as an employee on behalf of your employer or a postgraduate student of an affiliated institution which is paying any applicable article publishing charge ("APC") for Open Access articles. Where the Submitting Author wishes to make the Work available on an Open Access basis (and intends to pay the relevant APC), the terms of reuse of such Open Access shall be governed by a Creative Commons licence – details of these licences and which Creative Commons licence will apply to this Work are set out in our licence referred to above.

Other than as permitted in any relevant BMJ Author's Self Archiving Policies, I confirm this Work has not been accepted for publication elsewhere, is not being considered for publication elsewhere and does not duplicate material already published. I confirm all authors consent to publication of this Work and authorise the granting of this licence.

For peer review only

Observational Feasibility Study to Investigate the Quality of Life of Anticoagulated Participants with Atrial Fibrillation Following a Bleed

Hayley A Hutchings[1*], Kirsty Lanyon[1], Gail Holland[1], Raza Alikhan[2], Rhys Jenkins[3], Hamish Laing[4], Arfon Hughes[5], Trudie Lobban[6], Kevin G Pollock[7], Daniel Tod[1], Steven Lister[7]

1 Swansea University Medical School, Swansea University, Singleton Park, Swansea SA2 8PP, UK

*Corresponding author- Hayley A Hutchings

Email: h.a.hutchings@swansea.ac.uk

2 Department of Haematology, University Hospital of Wales, Cardiff and Vale University Health Board, UK

3 Swansea City GP Cluster, Swansea, UK

4 VBHC Academy, School of Management, Swansea University

5 Public and Patient Involvement Representative

6 Arrhythmia Alliance, Public and Patient Representative, UK

7 Bristol Myers Squibb, Uxbridge Business Park, Sanderson Road, Uxbridge, Middlesex UB8 1DH, UK

Running head: quality of life after a bleed

For peer review only - http://bmjopen.bmj.com/site/about/guidelines.xhtml

ABSTRACT

Objective

To evaluate the feasibility of recruiting participants diagnosed with atrial fibrillation (AF) taking oral anticoagulation therapies (OACs) and recently experiencing a bleed to collect quality of life (QoL) information.

Design

Observational feasibility study. The study aimed to determine the feasibility of recruiting participants with minor and major bleeds, the most appropriate route for recruitment and the appropriateness of the Patient Reported Outcome Measures (PROMs) selected for collecting quality of life information in AF patients, and the preferred format of the surveys.

Setting

Primary care, secondary care, and via an online patient forum.

Participants

The study population was adult patients (≥ 18) with Atrial Fibrillation (AF) taking oral anticoagulation therapies (OATs) who had experienced a recent major or minor bleed within the last four weeks.

Primary and Secondary outcome measures

Primary outcome:

Patient reported outcome measures (PROMs): EuroQol 5 dimensions-5 levels (EQ-5D-5L); Perception of anticoagulant treatment questionnaire, part 2 only (PACT-Q, part 2); Atrial fibrillation effect on quality of life (AFEQT)

Secondary outcomes:

Location of bleed; bleed severity; current treatment; patient perceptions of quality of life in relation to bleeding events.

Results

We recruited 32 participants to the study- 14 from primary care and 18 through the AF forum. No participants were recruited through secondary care. Despite 32 participants consenting, only 26 initial surveys were completed. We received follow-up surveys from 11 participants (8 primary care and 3 AF forum). COVID-19 had a major impact on the study.

Conclusions

Primary care was the most successful route for recruitment. Most participants recruited to the study experienced a minor bleed. Further ways to recruit in secondary care should be explored, especially to capture more serious bleeds.

Registration

For peer review only - http://bmjopen.bmj.com/site/about/guidelines.xhtml

The study was adopted onto the NIHR Portfolio (I.D. #47771) and registered with www.ClinicalTrials.gov (#NCT04921176) in February 2021.

Strengths and limitations of this study

- Multiple routes were used to identify potential participants with AF who had experienced a recent bleed- primary care, secondary care and through an online AF forum.
- Participants were able to complete the survey online (via REDCap®), on a paper-based form or over the telephone.
- Three validated patient reported outcome measures (PROMs) were used to gather different aspects of the impact of bleeding on AF patients.
- COVID- 19 impacted on the recruitment of participants and alternative mechanisms for recruitment need to be considered.

Key words: anticoagulation; bleeding disorders & coagulopathies; feasibility studies; quality of life

Word count (excluding title page, abstract, references, figures, tables or acknowledgments)- 3656

For peer review only

For peer review only - http://bmjopen.bmj.com/site/about/guidelines.xhtml

INTRODUCTION

Atrial Fibrillation (AF) is the most common cardiac arrythmia (irregular heart rhythm disorder). Estimated overall prevalence has been reported to be between 0.4-1%(1, 2). UK estimates have suggested that AF prevalence is increasing and could be as high as 3.29%(3). Many clinicians however debate the true global prevalence, believing that it is likely to be underestimated as many individuals are asymptomatic and are likely to go undiagnosed(4). The rate increases with age over 60 years(5) and it is estimated to be much higher in individuals over 80 and in Western societies, with suggested rates as high as 14%(6). There are known to be high numbers of individuals with undiagnosed AF(7-9).

AF is a chronic condition associated with significant risks of thromboembolism, stroke, and mortality(10, 11). Oral anticoagulation therapies (OATs)(1) are normally prescribed to reduce the risks of thromboembolism and AF-related stroke, but are associated with the side effect of bleeding, which in some cases can be serious(10, 12, 13). These bleeding complications can include intracranial haemorrhage(14), gastrointestinal bleeding, and haematuria(15). Bleeding as a side effect of OATs(16) remains a major challenge for clinicians that can often result in non-adherence and/or resistance to therapy(17). While hospitalized AF-related stroke rates have declined and are significantly associated with increased anticoagulant uptake(18), the increased use of OATs has led to an increase in the number of bleeding events over time(10).

Patients are treated by healthcare providers with the primary goal of improving their health and well-being. Historically this improvement in health has been judged by improvement in biochemical, histological, radiological or clinical assessments. It is hypothesised that this approach does not always correlate with improvement from the patient perspective(19). From a patient perspective, improving health is reflected in the documentation of their symptoms and experience of healthcare provision, which are more appropriately collected directly from the patient(20). With a move towards shared-decision making and patient-centred care there is growing recognition within the healthcare community of the importance of the patient perspective and the need to consider patient-reported outcome measures (PROMs) as a key component of a holistic approach to patient care.

PROMs were initially developed for research use, and many regulatory authorities such as the European Medicines Agency (EMA) and the United States (US) Food and Drug Administration (FDA) advocate their use(21-23). The U.S. Food and Drug Administration (FDA) defines a PROM as “any report of the status of a patient’s health condition that comes directly from the patient, without

For peer review only - http://bmjopen.bmj.com/site/about/guidelines.xhtml

interpretation of the patient's response by a clinician or anyone else"(22). The European Medicines Agency state that "Any outcome evaluated directly by the patient himself and based on the patient's perception of a disease and its treatment(s) is called a patient-reported outcome (PRO)"(24). PROMs include quality of life (QoL) measures, disease severity scales, and patient experience measures.

The collection of PROMs aligns well with the increased drive within healthcare organisations for Value-Based healthcare, where organisations aim to achieve the best possible outcomes for patients with the available resources(25, 26). As more clinicians recognise the benefit of collecting PROMs in addition to measuring clinical outcomes, PROMs have seen an increased use in routine clinical practice(27).

AF is thought to have a detrimental effect on QoL in patients (28-33). This research has focused on exploring QoL following various treatments or procedures(34-36), the development of AF specific QoL measures(37-40), or the use of existing QoL measures in AF populations(41, 42). There is some evidence that bleeding events may have an adverse effect on patient QoL(43, 44) but there has been limited exploration of QoL following a bleed in AF patients(42, 45). The aim of this study was to determine the feasibility of collecting QoL information from anticoagulated AF participants who had recently experienced a bleed. If this proves feasible, we plan to embark on a fully-powered study to evaluate the different dimensions of QoL of AF patients following a bleed.

METHODS

Selection of PROM to assess QoL

Prior to identifying sites and recruitment of participants, we undertook a process of PROM selection in order to select suitable tools that would allow us to assess QoL in AF participants who had experienced a recent bleed (reported elsewhere). This included a review of the literature to identify generic and disease specific PROMs that had been used in AF participants. Following the compilation of a list of possible PROMs, we undertook a rigorous evaluation of the identified PROMs that included an assessment of the overall validity of the PROMs, their content validity in terms of appropriateness of questions relating to bleeding events with AF, and how widely the PROM had previously been used in AF. This was informed by existing literature(46, 47) and included a clear justification as to why the PROM was used(48). In selecting the PROMs, we were also mindful to ensure the burden to participants was kept to a minimum, so also explored the number of questions within relevant PROMs. We worked with the two Public and Patient Involvement representatives when assessing the PROMs for help in considering their usefulness to the study.

For peer review only - http://bmjopen.bmj.com/site/about/guidelines.xhtml

Following the PROM scoping process, we agreed on three PROMs for use in the study:

- Euroquol 5 Dimensions-5 Levels (EQ-5D-5L)(49),
- Perception of Anticoagulant Treatment Questionnaire, Part 2 only (PACT-Q, Part 2)(50, 51); and
- Atrial fibrillation Effect on QualiTy-of-life (AFEQT)(41).

Participant Recruitment

We recruited adult participants (aged ≥18 years) who had a diagnosis of AF and were actively prescribed OATs for their AF. We identified participants who had experienced a recent bleed, up to a maximum of 30 days prior to enrolment. No upper age limit was imposed, but participants had to fit all other inclusion and exclusion criteria.

Inclusion criteria

- Adult participants (≥18 years)
- Participants who had a fluent understanding of English and were able to comprehend all study information and literature to provide fully informed consent
- Atrial fibrillation (AF) as the primary diagnosis
- Had a major or minor bleed up to a maximum of 30 days prior to date of enrolment
- Prescribed oral anticoagulation for AF

Exclusion Criteria

- Pregnant women
- Participants with active cancer
- Participants unable to consent for themselves
- Participants on concomitant antiplatelet therapy

As the study was designed to test feasibility for a fully powered definitive study, we aimed to recruit between 50 and 80 participants in the first instance based on recommended sample size guidance(52). Figure 1 outlines the stages of the study.

For peer review only

For peer review only - http://bmjopen.bmj.com/site/about/guidelines.xhtml

Participating sites

For this feasibility study, we selected participants from the Swansea, UK area. We utilised three recruitment pathways for the study to test the feasibility of identifying and recruiting participants with minor and major bleeds:

- Primary care – Participants attending anticoagulation clinics of a large city General Practice cluster made up of 8 practices and serving almost 51,000 patients in Swansea, UK. Participants were identified by practice nurse/pharmacist as having a recent bleed.
- Secondary care – Participants admitted to the emergency department of a large UK University teaching hospital in Swansea, UK with a bleed were identified by research nurses/secondary care-based general practitioner.
- Direct participant – The study was publicised on the Arrhythmia Alliance website and newsletters (Arrhythmia Alliance UK (heartrhythmalliance.org)) and individuals who formed part of this online forum and database were invited to participate.

When participants were identified through primary and secondary care, they were provided with a study information sheet for their consideration. If the patient was willing to participate, they were asked to return the expression of interest form to the study researcher in the supplied freepost envelope. The study researcher then contacted participants to provide them with a 'study pack'. As we aimed to determine the best approach for collecting the QoL information, we offered the participants the option of an electronic link to a REDCap® survey, a paper survey sent to their home with a freepost envelope, or the option to complete the survey by telephone. Individuals from the AF forum were initially provided with a weblink to the REDCap® survey through the website. Latterly, they were given the option to contact the researcher and be supplied with a paper survey.

For peer review only - http://bmjopen.bmj.com/site/about/guidelines.xhtml

Participants Survey Pack

We collected basic demographics from participants along with details of any bleed(s) they had experienced. We asked all participants to complete three PROMs for the study survey at baseline: the Euroquol 5 Dimensions-5 Levels (EQ-5D-5L)(49), Perception of Anticoagulant Treatment Questionnaire (PACT-Q) Part 2(50, 51), and the Atrial fibrillation Effect on QualiTy-of-life (AFEQT)(41).

We estimated that completion of all study questionnaires would take no longer than 30 minutes. Participants who agreed to participate were asked to complete the survey at two time points - once following consent, and then again at 90±14 days post-enrolment. Participants who completed the survey digitally via the REDCap® system received a second online link to complete the survey; those who completed paper forms were sent a follow up via post (along with a stamped addressed envelope for ease of return); and those who completed over the phone were contacted via telephone to complete the follow-up. Participants were contacted 3 weeks prior to the 90-day follow-up date. If no reply was received within 2 weeks of first correspondence, a reminder letter (with a second copy of all questionnaires) was sent, or a follow-up email or telephone call was made.

Data analysis

Quantitative data were checked, cleaned, and collated in REDCap® prior to being transferred to Stata Version 17.0.for analyses. As this was a feasibility study and not powered to detect differences between groups/OATs, no formal statistical comparisons were made. Data are presented descriptively as number, percentages, or means (standard deviation). QoL scores were calculated according to developers' guidance. Descriptive QoL scores are reported for AF participants following a minor or major bleed(53) while anticoagulated and by recruitment route. Categorisation of the bleed into minor or major was undertaken by a clinical professional (RA).

Ethics

We complied with Good Clinical Practice (GCP) guidelines and the General Data Protection Regulation (GDPR) in the conduct of the study and the collection of participant information. The study was given ethical approval from the London - Riverside Research Ethics Committee Research Ethics Committee (IRAS ID 279646), and Swansea Bay University Health Board R&D. The study research governance sponsor was Swansea University.

Participants were required to provide consent before enrolment into the study. Prior to consent we provided participants with an information sheet which ensured that they were clearly and fully

For peer review only

For peer review only - http://bmjopen.bmj.com/site/about/guidelines.xhtml

informed about the purpose of the study, potential risks, use of their data, and their rights and responsibilities when participating in the study. They were also told that they had the right to withdraw at any point in the study. Participants who completed the survey via the REDCap® system consented to the study electronically. Participants completing paper forms were required to complete a paper consent and we obtained verbal consent for those completing over the telephone. We asked participants for informed consent for their anonymised data and interview quotes (where relevant) to be used in any future publications.

We were aware that participants may have experienced low mood or anxiety associated with their AF. If a participant reported, or showed signs of low mood, distress or anxiety, we encouraged them to discuss this with their primary or secondary care researcher and the participant was signposted to local relevant services or advised to contact their GP.

We stored all data securely within the password protected REDCap® secure web application while the study was conducted, and subsequently anonymised for analysis purposes.

COVID implications/changes

This study was originally conceived and designed before the COVID-19 pandemic. Shortly after receiving ethical approval and prior to the start of recruitment, the country went into its first lockdown to prevent onward transmission of the virus. Initially, the study was paused as we knew that participant recruitment through primary and secondary care would be impacted. As time progressed, however, we decided to adapt the study to take account of the situation and the change in operating practices across sites. The initial protocol included processes for face-to-face recruitment in primary care (through routine anticoagulation clinics) and in secondary care (through identification of AF bleeding cases through medical records and approach of patients on the wards).

To minimise any potential COVID-19 risk to both research staff and patients, it was agreed therefore that the study design should operate in line with current guidelines set out by the UK and Welsh Government for COVID-19 management. Up-to-date Government guidance was therefore followed throughout the study. This had a direct consequence on some aspects of study conduct. Study visits were therefore changed to ensure that they could be conducted remotely, until such time where face-to-face visits were permitted. Key members of the study team monitored relevant guidance for GP surgeries and hospitals closely and necessary adjustments to study conduct were made accordingly, and consistently with any new information and/or restrictions as it was released. Where study tasks

For peer review only - http://bmjopen.bmj.com/site/about/guidelines.xhtml

were conducted remotely, Health Research Authority (HRA) advice released for conducting research during the COVID-19 pandemic was followed, where applicable. Any study visits conducted in person took place with all required precautions in place to help prevent the spread of the virus.

RESULTS

We received initial expressions of interest from 85 participants in primary care. These expressions of interest were however not translated into participation in most cases. 31 questionnaires were sent out following successful initial contact with prepaid addressed envelopes for return but no further contact with the participant could be made. In addition, 18 participants started the survey on the AF forum. A total of 32 participants consented to participate in the study - 14 from primary care and 18 through the AF forum. No participants were recruited through secondary care.

COVID-19 had a major impact on the study, particularly in secondary care. We had difficulty recruiting in secondary care for three reasons. Firstly, many research nurses were transferred back to help manage frontline NHS activities; secondly, those who were still active had difficulty gaining access to participants and wards during lockdown; and thirdly, Welsh Government mandated that research nurse activity should be prioritised to COVID-19 vaccine studies. As a result, we focused on the two other recruitment routes, primary care and the AF forum.

Despite 32 participants consenting to participate, only 26 initial surveys were completed (14 from primary care and 12 from the AF forum), with 6 from the AF forum being abandoned prior to completion of any questions on REDCap®. We contacted primary care contacts at least twice if we had received an expression of interest from them.

We received follow-up surveys from 11 participants (8 primary care and 3 AF forum). Figure 2 illustrates the numbers of participants expressing an interest and subsequently consenting to participate in the study.

Table 1 illustrates the responses to the general questions from the 26 participants in the primary care and AF forum groups. We only had demographic information for the primary care group, where clinical data were cross referenced to patient records. The primary care patients were all white and mostly female. The HASBLED score indicated that participants were at low risk of bleeding.

For peer review only - http://bmjopen.bmj.com/site/about/guidelines.xhtml

In both the primary care and AF forum groups, most participants had pre-existing conditions. There were a range of times since the bleed, including beyond 30 days, despite recruitment aiming to focus on bleeds of 30 days or less. Minor bleeding was more common than major bleeding in both groups, with 6/14 (42.9%) of participants in the primary care group and 5/12 (41.7%) in the AF forum group indicating that they had fairly frequent minor bleeds (1-2 per week or 1-2 per month). This was in contrast to the frequency of major bleeds, where 10/14 (71.4%) of participants from primary care and 7/12 (58.3%) from the AF forum indicated that they almost never had major bleeds. Clinical classification of the most recent bleed (where documented) indicated that there were 18 minor, 2 major and 1 unknown.

Table 2 illustrates the QoL scores from the three questionnaires. The EQ5D index scores range between 0 and 100, with a higher index score indicating better QoL. The mean EQ5D index score in the primary care group was lower than that in the AF forum group. The follow up score in the primary care group was higher, but still less than the AF forum group.

The disease specific AFEQT overall and subscale scores range from 0 to 100. A score of 0 corresponds to complete disability, while a score of 100 corresponds to no disability. The AFEQT overall and subscale scores were largely in the positive range indicating limited disability due to AF. The overall, symptom sub-scale and daily activities sub-scales showed little change between the first and follow-up survey in the primary care and AF forum groups. In terms of the treatment concern sub-scale, there was a deterioration in the primary care group and an improvement in the AF forum group at follow-up. Both groups had a deterioration in response to the two AFEQT satisfaction questions (Q19 and 20, see Table 2). These questions relate to how well they think their current treatment controls their AF and the extent to which their treatment has relieved their symptoms.

The PACT-Q Part 2 scores range from 0 to 100, for the convenience and satisfaction domains, with a higher score indicating higher convenience and higher satisfaction. The convenience scores were comparable between the primary care and AF forum groups at the initial survey and changed very little at three months. The initial satisfaction scores were again comparable across the two groups, but the primary care group showed a deterioration at 3 months, whereas the AF forum group showed an improvement in scores after 3 months.

DISCUSSION

For peer review only - http://bmjopen.bmj.com/site/about/guidelines.xhtml

After receiving the necessary ethics and R&D approvals for the study, the UK went into lockdown and we were forced to initially pause the study. When we were in a position to restart, we had to modify the study to take account of changing healthcare practices. This included remote engagement and varying formats for completion of the questionnaires. Recruitment in secondary care was mostly impacted and we were unable to recruit any patients through this route. This was largely due to nurses being reassigned to front-line roles, limited access to wards, and a focus on recruitment to Covid-19 studies. We were still able to recruit through primary care and the AF online forum. There were however a number of incomplete questionnaires submitted through the online forum. This may indicate that patients find online forms difficult to complete(54) or that they were unsure of the relevance of the questions or found the survey too long. Separate qualitative interviews with patients will explore these concepts further.

Primary care therefore appeared to be the best way to recruit participants. This may have been because they were introduced to the study by the primary care pharmacist whom they knew and they were therefore not contacted 'cold'. We received initial expressions of interest in primary care from 90 participants. This did not however translate into 90 completed questionnaires. This may be because of the time lapse between discussing the study and subsequently being contacted by the researcher or misunderstanding regarding the rationale for the study. Had the participants received the questionnaire on initial contact, this may have increased the completion rate.

Most of the patients that completed the survey had minor bleeds. This may be due to the fact that most patients were recruited through primary care. Had we recruited through secondary care, we may have seen more major bleeds.

Selecting the PROMs for the study was challenging. The focus of the study was to test the feasibility of collecting data regarding quality of life in AF participants on OATs who experienced a bleed. Details of the selection process for the PROMs will be reported separately, but the final decision was based on 1) previous use of the PROMs with the AF population; 2) assessment of the validity of the PROMs; and 3) relevance of the questions to a bleeding event. There were few PROMs that had questions that specifically focused on the bleed. This may have also contributed to the fact that the response rate was lower than expected.

It was difficult to interpret the quality of life scores as the study was not powered to detect any statistically significant differences. In addition, there was a great deal of variability in the scores across

For peer review only - http://bmjopen.bmj.com/site/about/guidelines.xhtml

the different scales even within the same groups. Due to the limited number of participants recruited and the variability in the scores obtained, contextualising these scores with existing literature is therefore difficult.

Further work needs to be undertaken to explore alternative ways of recruiting through secondary care. In our study we utilised research nurses with the aim of accessing ward admission records to identify potential patients. This mechanism for recruitment was particularly impacted by the pandemic; access to the wards was restricted, many research nurses were moved back to frontline activities, and covid-19 research studies were prioritised. Recruitment through secondary care may be improved by working directly with clinicians who manage AF patients on OATs. It is likely that this would also increase the number of participants recruited with major bleeds and help to determine the most appropriate time to approach participants, especially as they may be too ill during their inpatient episode.

The way we recruited through primary care could also be streamlined, with participants being invited to participate following their initial contact with the lead clinician managing the bleeding clinics rather than being contacted by the researcher following completion of an expression of interest. The relevance of the questionnaires may have impacted on the completion of the survey through the online forum, so use of a more specific PROM that focused on bleeding issues may facilitate this.

Despite the challenges of undertaking the study in the pandemic, we were still able to recruit 32 patients. A further fully- powered study is now needed to explore whether quality of life is impacted by bleeding episodes in AF patients on OATs, and whether the extent of the bleed has an effect on quality of life.

AUTHOR CONTRIBUTIONS

HAH and SL conceived the study. HAH designed the initial study proposal, led on interpretation of the findings and drafted the manuscript. KL was responsible for site set-up, ethical approvals, participant recruitment and data collection; participated in interpretation of findings and provided input on the manuscript. GH provided overall study governance support, specifically in relation to ethical aspects, provided input into final protocol, participated in interpretation of findings and provided input on the manuscript. RA provided input into final protocol, was responsible for clinical interpretation of bleeds, participated in interpretation of findings and provided input on the manuscript. RJ led primary care recruitment, provided input into final protocol, participated in interpretation of findings and provided input on the manuscript. TL led AF forum recruitment, provided input into final protocol, provided

For peer review only - http://bmjopen.bmj.com/site/about/guidelines.xhtml

For peer review only

PPI input on survey design, participated in interpretation of findings and was provided input on the manuscript. AH provided input into final protocol, provided PPI input on survey design, participated in interpretation of findings and provided input on the manuscript. DT was responsible for writing the analysis plan, final analysis and interpretation of the findings and provided input on the manuscript. HL, KP provided input into final protocol, participated in interpretation of findings and provided input on the manuscript. All authors reviewed and approved the final manuscript.

ACKNOWLEDGEMENTS

We would like to thank all participants for completing the survey, the general practice nurses from Swansea Bay City Cluster who helped with recruitment, and the Arrhythmia Alliance for hosting the online survey.

FUNDING AND GOVERNANCE

The Chief Investigator of the study was Professor Hayley Hutchings, Swansea Trials Unit, Swansea University (SU). Under the UK Policy Framework for Health and Social Care Research SU met the requirements of research governance sponsor. BMS/Pfizer funded the research and under the UK policy framework were part of a partnership that accepted overall responsibility for proportionate and effective arrangements to be in place to set up, run and report the research project. They were represented on the Trial Steering Committee throughout the project.

CONFLICT OF INTEREST

Hayley A Hutchings leads this independent research which was funded by Pfizer and Bristol Myers Squibb.

Hamish Laing is an employee of Swansea University who has received a grant for a Value-Based Healthcare programme of work from Pfizer Ltd., which he leads and support to attend a meeting.

Steven Lister was an employee of BMS at the time of the study and holds stock in BMS and Pfizer.

Kevin Pollock is an employee of BMS and holds stock in BMS.

Gail Holland, Kirsty Lanyon, Arfon Hughes, Rhys Jenkins, Trudie Lobban, declare that they have no conflict of interest.

DATA SHARING AGREEMENT

Statistical code, and anonymised dataset will be made available on the Zenodo open access data repository.

For peer review only - http://bmjopen.bmj.com/site/about/guidelines.xhtml

REFERENCES

1. Liu GJ, Wang YF, Chen PY, Chang W, Tu ML, Chang LY, et al. The efficacy and safety of novel oral anticoagulants for the preventive treatment in atrial fibrillation patients: a systematic review and meta-analysis. Drug Deliv. 2014;21(6):436-52.
2. Zulkifly H, Lip GYH, Lane DA. Epidemiology of atrial fibrillation. Int J Clin Pract. 2018;72(3):e13070.
3. Adderley NJ, Ryan R, Nirantharakumar K, Marshall T. Prevalence and treatment of atrial fibrillation in UK general practice from 2000 to 2016. Heart. 2019;105(1):27-33.
4. Patel NJ, Atti V, Mitrani RD, Viles-Gonzalez JF, Goldberger JJ. Global rising trends of atrial fibrillation: a major public health concern. Heart. 2018;104(24):1989-90.
5. Darby-Stewart A, Dachs R, Graber MA. Rivaroxaban vs. warfarin for stroke prevention in patients with nonvalvular atrial fibrillation. Am Fam Physician. 2012;85(6):577-86.
6. Kodani E, Atarashi H. Prevalence of atrial fibrillation in Asia and the world. Journal of Arrhythmia. 2012;28(6):330-7.
7. Turakhia MP, Shafrin J, Bognar K, Trocio J, Abdulsattar Y, Wiederkehr D, et al. Estimated prevalence of undiagnosed atrial fibrillation in the United States. PLoS One. 2018;13(4):e0195088.
8. Clua-Espuny JL, Lechuga-Duran I, Bosch-Princep R, Roso-Llorach A, Panisello-Tafalla A, Lucas-Noll J, et al. Prevalence of undiagnosed atrial fibrillation and of that not being treated with anticoagulant drugs: the AFABE study. Rev Esp Cardiol (Engl Ed). 2013;66(7):545-52.
9. Suissa L, Lachaud S, Mahagne MH. Optimal timing and duration of continuous electrocardiographic monitoring for detecting atrial fibrillation in stroke patients. J Stroke Cerebrovasc Dis. 2013;22(7):991-5.
10. Hansen PW, Sehested TSG, Fosbol EL, Torp-Pedersen C, Kober L, Andersson C, et al. Trends in warfarin use and its associations with thromboembolic and bleeding rates in a population with atrial fibrillation between 1996 and 2011. PLoS One. 2018;13(3):e0194295.
11. Kannel WB, Wolf PA, Benjamin EJ, Levy D. Prevalence, incidence, prognosis, and predisposing conditions for atrial fibrillation: population-based estimates. Am J Cardiol. 1998;82(8A):2N-9N.
12. Turagam MK, Parikh V, Afzal MR, Gopinathannair R, Lavu M, Kanmanthareddy A, et al. Replacing warfarin with a novel oral anticoagulant: Risk of recurrent bleeding and stroke in patients with warfarin ineligible or failure in patients with atrial fibrillation (The ROAR study). J Cardiovasc Electrophysiol. 2017;28(8):853-61.
13. Lamberts M, Staerk L, Olesen JB, Fosbol EL, Hansen ML, Harboe L, et al. Major Bleeding Complications and Persistence With Oral Anticoagulation in Non-Valvular Atrial Fibrillation: Contemporary Findings in Real-Life Danish Patients. J Am Heart Assoc. 2017;6(2).
14. Hankey GJ. Intracranial hemorrhage and novel anticoagulants for atrial fibrillation: what have we learned? Curr Cardiol Rep. 2014;16(5):480.
15. Wallis CJD, Juvet T, Lee Y, Matta R, Herschorn S, Kodama R, et al. Association Between Use of Antithrombotic Medication and Hematuria-Related Complications. JAMA. 2017;318(13):1260-71.
16. Khan F, Huang H, Datta YH. Direct oral anticoagulant use and the incidence of bleeding in the very elderly with atrial fibrillation. J Thromb Thrombolysis. 2016;42(4):573-8.
17. Dar T, Yarlagadda B, Vacek J, Dawn B, Lakkireddy D. Management of Stroke risk in atrial fibrillation patients with bleeding on Oral Anticoagulation Therapy-Role of Left Atrial Appendage Closure, Octreotide and more. J Atr Fibrillation. 2017;10(4):1729.
18. Cowan JC, Wu J, Hall M, Orlowski A, West RM, Gale CP. A 10 year study of hospitalized atrial fibrillation-related stroke in England and its association with uptake of oral anticoagulation. Eur Heart J. 2018;39(32):2975-83.
19. Pakhomov SV, Jacobsen SJ, Chute CG, Roger VL. Agreement between patient-reported symptoms and their documentation in the medical record. Am J Manag Care. 2008;14(8):530-9.
20. Hutchings HA, Alrubaiy L. Patient-Reported Outcome Measures in Routine Clinical Care: The PROMise of a Better Future? Dig Dis Sci. 2017;62(8):1841-3.

For peer review only

For peer review only - http://bmjopen.bmj.com/site/about/guidelines.xhtml

21. European Medicines Agency. Reflection paper on th regulatory guidance for the use of Health-Related Quality of Life (HRQL) measures in the evaluation of medicinal products. Available from: https://www.ema.europa.eu/en/documents/scientific-guideline/reflection-paper-regulatory-guidance-use-healthrelated-quality-life-hrql-measures-evaluation_en.pdf 2005 [
22. U.S. Department of Health and Human Services, Food and Drug Administration, Center for Drug Evaluation and Research (CDER), Center for Biologics Evaluation and Research (CBER), Center for Devices and Radiological Health (CDRH). Guidance for Industry Patient-Reported Outcome Measures: Use in Medical Product Development to Support Labeling Claims. Available from: https://www.fda.gov/downloads/Drugs/GuidanceComplianceRegulatoryInformation/Guidances/UCM193282.pdf 2009 [
23. Revicki DA, Regulatory I, Patient-Reported Outcomes Task Force for the International Society for Quality of Life R. FDA draft guidance and health-outcomes research. Lancet. 2007;369(9561):540-2.
24. Downing J, Namisango E, Harding R. Outcome measurement in paediatric palliative care: lessons from the past and future developments. Ann Palliat Med. 2018;7(Suppl 3):S151-S63.
25. Porter ME, Larsson S, Lee TH. Standardizing Patient Outcomes Measurement. N Engl J Med. 2016;374(6):504-6.
26. Porter ME, Lee TH. The Strategy That Will Fix Health Care. Harvard Bus Rev. 2013;91(12):24-.
27. Black N. Patient reported outcome measures could help transform healthcare. BMJ. 2013;346:f167.
28. Peinado R, Arribas F, Ormaetxe JM, Badia X. Variation in quality of life with type of atrial fibrillation. Rev Esp Cardiol. 2010;63(12):1402-9.
29. Zhang L, Gallagher R, Neubeck L. Health-related quality of life in atrial fibrillation patients over 65 years: A review. Eur J Prev Cardiol. 2015;22(8):987-1002.
30. Deaton C, Dunbar SB, Moloney M, Sears SF, Ujhelyi MR. Patient experiences with atrial fibrillation and treatment with implantable atrial defibrillation therapy. Heart Lung. 2003;32(5):291-9.
31. Aliot E, Botto GL, Crijns HJ, Kirchhof P. Quality of life in patients with atrial fibrillation: how to assess it and how to improve it. Europace. 2014;16(6):787-96.
32. Ong L, Cribbie R, Harris L, Dorian P, Newman D, Mangat I, et al. Psychological correlates of quality of life in atrial fibrillation. Qual Life Res. 2006;15(8):1323-33.
33. Thrall G, Lane D, Carroll D, Lip GY. Quality of life in patients with atrial fibrillation: a systematic review. Am J Med. 2006;119(5):448 e1-19.
34. Pokorney SD, Kim S, Thomas L, Fonarow GC, Kowey PR, Gersh BJ, et al. Cardioversion and subsequent quality of life and natural history of atrial fibrillation. Am Heart J. 2017;185:59-66.
35. Sun Y, Zhu J, Ma C, Liu S, Yang Y, Hu D. Stroke Risk Status, Anticoagulation Treatment, and Quality-of-Life in Chinese Patients with Atrial Fibrillation: China Registry of Atrial Fibrillation (CRAF). Cardiovasc Ther. 2019;2019:7372129.
36. Bai Y, Bai R, Wu JH, Zhang T, Liu N, Shi XB, et al. Differences in Quality of Life Between Atrial Fibrillation Patients with Low Stroke Risk Treated With and Without Catheter Ablation. J Am Heart Assoc. 2015;4(9):e002130.
37. Coyne KS, Edvardsson N, Ryden A. Development and Validation of the AFImpact: An Atrial Fibrillation-Specific Measure of Patient-Reported Health-Related Quality of Life. Value Health. 2017;20(10):1355-61.
38. Badia X, Arribas F, Ormaetxe JM, Peinado R, de Los Terreros MS. Development of a questionnaire to measure health-related quality of life (HRQoL) in patients with atrial fibrillation (AF-QoL). Health Qual Life Outcomes. 2007;5:37.
39. Arribas F, Ormaetxe JM, Peinado R, Perulero N, Ramirez P, Badia X. Validation of the AF-QoL, a disease-specific quality of life questionnaire for patients with atrial fibrillation. Europace. 2010;12(3):364-70.

For peer review only

For peer review only - http://bmjopen.bmj.com/site/about/guidelines.xhtml

40. Medin J, Arbuckle R, Abetz L, Halling K, Kulich K, Edvardsson N, et al. Development and validation of the AFSymp: an atrial fibrillation-specific measure of patient-reported symptoms. Patient. 2014;7(3):319-27.
41. Spertus J, Dorian P, Bubien R, Lewis S, Godejohn D, Reynolds MR, et al. Development and validation of the Atrial Fibrillation Effect on QualiTy-of-Life (AFEQT) Questionnaire in patients with atrial fibrillation. Circ Arrhythm Electrophysiol. 2011;4(1):15-25.
42. Wang K, Li H, Kwong WJ, Antman EM, Ruff CT, Giugliano RP, et al. Impact of Spontaneous Extracranial Bleeding Events on Health State Utility in Patients with Atrial Fibrillation: Results from the ENGAGE AF-TIMI 48 Trial. J Am Heart Assoc. 2017;6(8).
43. Res LCS, Lubberts B, Shah SH, DiGiovanni CW. Health-related quality of life after adverse bleeding events associated with antithrombotic drug therapy - A systematic review. Hellenic J Cardiol. 2019;60(1):3-10.
44. Radaideh KM, Matalqah LM. Health-related quality of life among atrial fibrillation patients using warfarin therapy. Epidemiology Biostatistics and Public Health. 2018;15(1):e12763-1.
45. De Caterina R, Bruggenjurgen B, Darius H, Kohler S, Lucerna M, Pecen L, et al. Quality of life and patient satisfaction in patients with atrial fibrillation on stable vitamin K antagonist treatment or switched to a non-vitamin K antagonist oral anticoagulant during a 1-year follow-up: A PREFER in AF Registry substudy. Arch Cardiovasc Dis. 2018;111(2):74-84.
46. Kotecha D, Ahmed A, Calvert M, Lencioni M, Terwee CB, Lane DA. Patient-Reported Outcomes for Quality of Life Assessment in Atrial Fibrillation: A Systematic Review of Measurement Properties. PLoS One. 2016;11(11):e0165790.
47. Reynolds MR, Ellis E, Zimetbaum P. Quality of life in atrial fibrillation: measurement tools and impact of interventions. J Cardiovasc Electrophysiol. 2008;19(7):762-8.
48. Vibeke Høgh V, Delmar C, Riahi S, Kirsten Frederiksen K. Quality of Life - Exemplified through Patients Living with Atrial Fibrillation. J Nurs Care 2014; 3(2).
49. EQ-5D. EQ-5D-5L. About. Available from: https://euroqol.org/eq-5d-instruments/eq-5d-5l-about/ (Access date 13 January 2022) 2009 [
50. Prins MH, Guillemin I, Gilet H, Gabriel S, Essers B, Raskob G, et al. Scoring and psychometric validation of the Perception of Anticoagulant Treatment Questionnaire (PACT-Q). Health Qual Life Outcomes. 2009;7:30.
51. Prins MH, Marrel A, Carita P, Anderson D, Bousser MG, Crijns H, et al. Multinational development of a questionnaire assessing patient satisfaction with anticoagulant treatment: the 'Perception of Anticoagulant Treatment Questionnaire' (PACT-Q). Health Qual Life Outcomes. 2009;7:9.
52. Lancaster GA, Dodd S, Williamson PR. Design and analysis of pilot studies: recommendations for good practice. J Eval Clin Pract. 2004;10(2):307-12.
53. Schulman S, Kearon C, Subcommittee on Control of Anticoagulation of the S, Standardization Committee of the International Society on T, Haemostasis. Definition of major bleeding in clinical investigations of antihemostatic medicinal products in non-surgical patients. J Thromb Haemost. 2005;3(4):692-4.
54. Calvert MJ, Cruz Rivera S, Retzer A, Hughes SE, Campbell L, Molony-Oates B, et al. Patient reported outcome assessment must be inclusive and equitable. Nat Med. 2022;28(6):1120-4.

For peer review only

For peer review only - http://bmjopen.bmj.com/site/about/guidelines.xhtml

FIGURES

For peer review only

For peer review only - http://bmjopen.bmj.com/site/about/guidelines.xhtml

Figure 1. Flow chart of the stages involved in the study

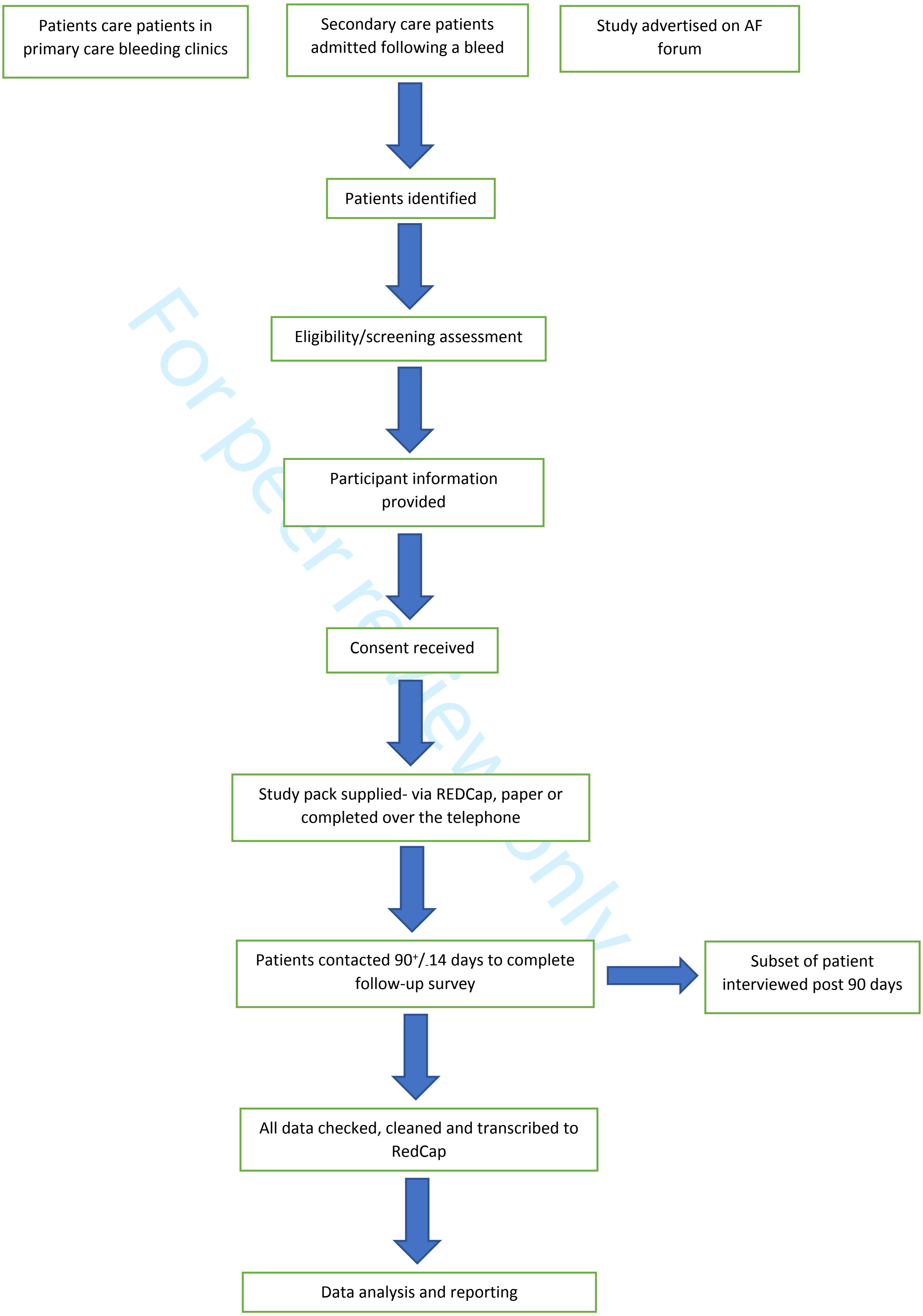

For peer review only - http://bmjopen.bmj.com/site/about/guidelines.xhtml

Figure 2. Flow chart of the participants recruited to the study and subsequently analysed

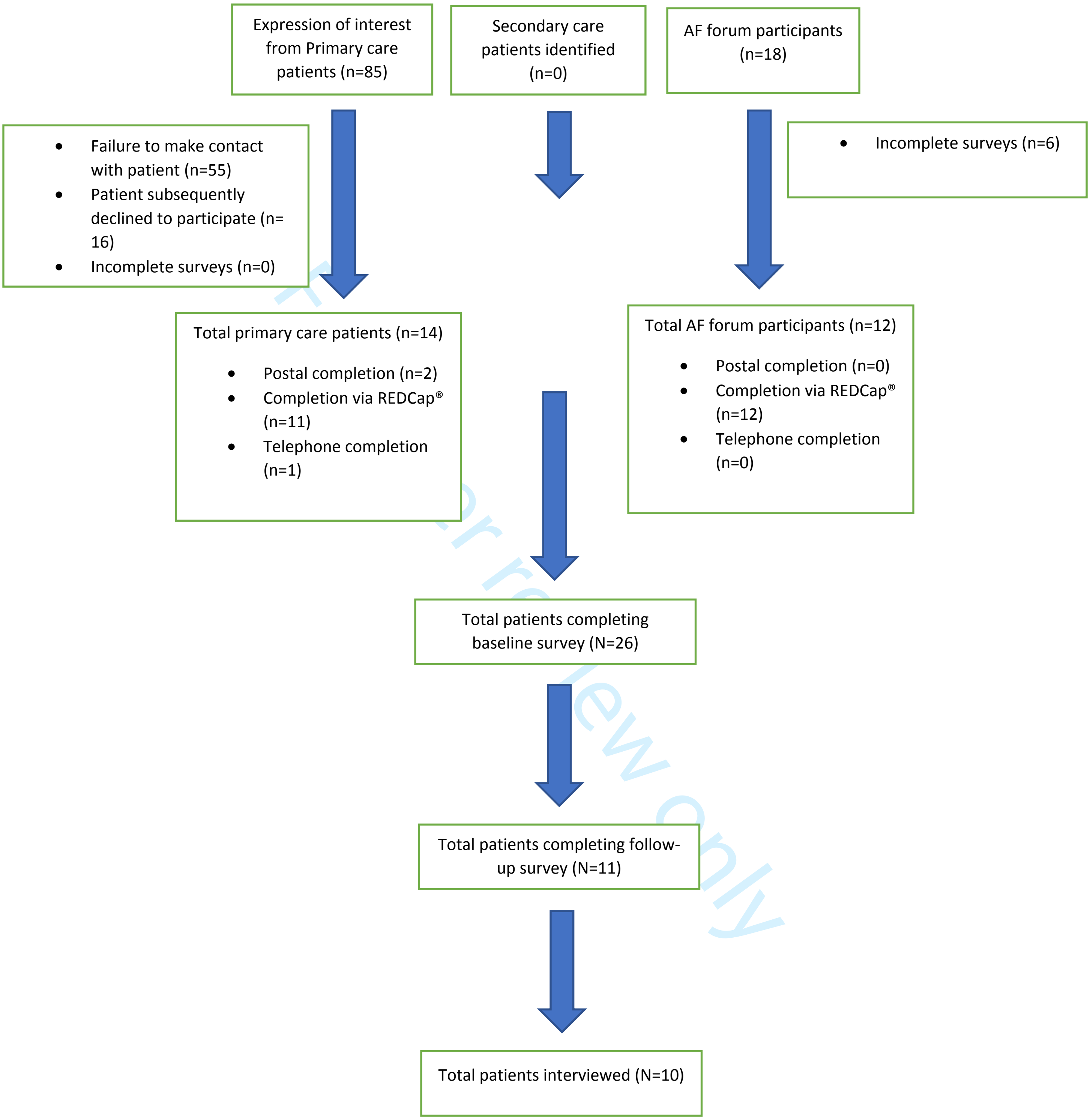

For peer review only - http://bmjopen.bmj.com/site/about/guidelines.xhtml

TABLES

For peer review only

For peer review only - http://bmjopen.bmj.com/site/about/guidelines.xhtml

Table 1. Demographics of the recruited participants

	Primary care (n=14)	AF forum (n=12)*
Ethnicity		
White	14(100%)	n/a
Sex (M:F)	3:11	n/a
CHA_2DS_2-VASc		
0	1	n/a
1	2	
2	1	
3	0	
4	5	
5	3	
6	2	
HAS-BLED		
0	1(7.1%)	n/a
1	5(35.7%)	
2	8(57.1%)	
Medication		
Apixaban	7(50%)	1(8.33%)
Dabigatran	0(0%)	1(8.33%)
Rivaroxaban	5(35.7%)	4(33.33%)
Warfarin	1(7.1%)	2(16.67%)
Xarelto	0(0%)	1(8.33%)
Missing	1(7.1%)	3(25%)
Co-morbidities/pre-existing disease		
Congestive heart failure	0	1
Hypertension	6	5
Hypotension	1	0
Ischaemic heart disease	0	1
Chronic Obstructive Pulmonary Disease	2	0
Diabetes related heart disease	1	1
Other	9	3
Days since bleed		
0-7	3(21.4%)	2(16.66%)
8-30	0(0%)	2(16.67)
31-60	1(7.1%)	4(33.33%)
60+	1(7.1%)	0(0%)
Unknown	9(64.3%)	4(33.33%)
Location of Bleed		
Internal	3(21.4%)	4(33.33%)
External	7(50%)	4((33.33%)
Unsure	1(7.1%)	1(8.33%)
Missing	3(21.4%)	3(25%)
Body location of external bleed		
Head	0(0%)	2(50%)
Neck	1(14.3%)	0(0%)
Forearm	2(28.6%)	0(0%)
Calf/shin	1(14.3%)	1(25%)

For peer review only

Feet, ankle, toes	1(14.3%)	0(0%)
Haemorrhoids	1(14.3%)	0(0%)
Missing	1(14.3%)	1(25%)
Body location of internal bleed		
Head	2(66.67%)	0 (0%)
Respiratory system	0(0%)	1(25%)
Reproductive system	0(0%)	1(25%)
Other	1(33.33%)	2(50%)
Frequency of minor bleeds		
1-2 times per week	4(28.6%)	1(8.33%)
1-2 times per month	2(14.3%)	4(33.33%)
1-2 times over 6 months	3(21.4%)	2(16.67%)
1-2 times a year	0(0%)	1(8.33%)
Almost never	3(21.4%)	2(16.67%)
Missing	2(14.3%)	2(16.67%)
Frequency of major bleeds		
1-2 per week	1(7.1%)	0(0%)
1-2 times a year	0(0%)	2(16.67%)
Almost never	10(71.4%)	7(58.33%)
Missing	3(21.4%)	3(25%)

- Demographic/symptom information was not collected on the AF forum as it could not be clinically verified.

For peer review only

For peer review only - http://bmjopen.bmj.com/site/about/guidelines.xhtml

Table 2. Quality of life scores at baseline and follow up split by primary care and AF forum patients measured using the EQ5D, AFEQT and PACT questionnaires.

a) EQ5D scores

EQ-5D UK Index*						
Mean (Min, Max)	First Survey		Follow-up Survey		Total	
Primary Care	n=13	0.470 (-0.200, 1.000)	n=8	0.694 (0.393, 1.000)	n=21	0.629 (-0.043, 1.000)
Online Forum	n=12	0.809 (0.476, 1.000)	n=3	0.893 (0.679, 1.000)	n=15	0.870 (0.568, 1.000)
Total	n=25	0.633 (-0.200, 1.000)	n=11	0.748 (0.393, 1.000)	n=36	0.729 (-0.043, 1.000)

* The index has a score range of -0.285 – 1.

b) AFEQT Scores

AFEQT		
Mean (Min, Max)	First Survey	Follow-up Survey
Overall Score		
Primary Care	64.8 (18.5, 98.1)	63.3 (37.0, 91.7)
AF Forum	65.7 (25.9, 94.4)	77.2 (50.5, 93.5)
Symptom subscale		
Primary Care	76.4 (33.3, 100)	74.0 (37.5, 95.8)
AF Forum	78.0 (37.5, 100)	81.9 (58.3, 95.8)
Daily Activities Subscale		
Primary Care	50.0 (14.6, 100)	59.8 (8.3, 95.8)
AF Forum	58.7 (10.4, 91.7)	76.4 (47.9, 95.8)
Treatment concern Subscale		
Primary Care	76.9 (13.9, 100)	59.1 (11.1, 83.3)
AF Forum	66.9 (33.3, 91.4)	75.0 (50.0, 91.7)

AFEQT Satisfaction Questions		
Mean (Min, Max)	First Survey	Follow-up Survey
Q19. How well your current treatment controls your atrial fibrillation?		
Primary Care	75.0 (50.0, 100)	62.5 (16.7, 100)
AF Forum	65.3 (0.0, 83.3)	61.1 (50.0, 66.7)
Q20. The extent to which treatment has relieved your symptoms of atrial fibrillation?		
Primary Care	73.6 (50.0, 100)	62.5 (16.7, 83.3)
AF Forum	66.7 (0.0, 100)	61.1 (50.0, 66.7)

c) PACT-Q Part 2 Scores

PACT-Q Scores		
Mean (Min, Max)	First Survey	Follow-up Survey
Convenience score		
Primary Care	87.0 (59.6, 100)	86.8 (69.2, 100)

For peer review only - http://bmjopen.bmj.com/site/about/guidelines.xhtml

AF Forum	86.5 (65.4, 98.1)	86.5 (76.9, 98.1)
Satisfaction score		
Primary Care	65.4 (39.3, 96.4)	61.6 (50.0, 78.6)
AF Forum	67.5 (39.3, 89.3)	76.2 (64.3, 92.9)

For peer review only

For peer review only - http://bmjopen.bmj.com/site/about/guidelines.xhtml

STROBE Statement—Checklist of items that should be included in reports of ***cross-sectional studies***

	Item No	Recommendation	Page No
**Title and abstract**	1	(*a*) Indicate the study's design with a commonly used term in the title or the abstract	1
		(*b*) Provide in the abstract an informative and balanced summary of what was done and what was found	2-3
**Introduction**			
Background/rationale	2	Explain the scientific background and rationale for the investigation being reported	5
Objectives	3	State specific objectives, including any prespecified hypotheses	5
**Methods**			
Study design	4	Present key elements of study design early in the paper	8
Setting	5	Describe the setting, locations, and relevant dates, including periods of recruitment, exposure, follow-up, and data collection	7
Participants	6	(*a*) Give the eligibility criteria, and the sources and methods of selection of participants	6
Variables	7	Clearly define all outcomes, exposures, predictors, potential confounders, and effect modifiers. Give diagnostic criteria, if applicable	8
Data sources/ measurement	8*	For each variable of interest, give sources of data and details of methods of assessment (measurement). Describe comparability of assessment methods if there is more than one group	*8*
Bias	9	Describe any efforts to address potential sources of bias	7
Study size	10	Explain how the study size was arrived at	8
Quantitative variables	11	Explain how quantitative variables were handled in the analyses. If applicable, describe which groupings were chosen and why	8
Statistical methods	12	(*a*) Describe all statistical methods, including those used to control for confounding	8
		(*b*) Describe any methods used to examine subgroups and interactions	N/A
		(*c*) Explain how missing data were addressed	8
		(*d*) If applicable, describe analytical methods taking account of sampling strategy	N/A
		(*e*) Describe any sensitivity analyses	N/A
**Results**			
Participants	13*	(a) Report numbers of individuals at each stage of study—eg numbers potentially eligible, examined for eligibility, confirmed eligible, included in the study, completing follow-up, and analysed	10-11
		(b) Give reasons for non-participation at each stage	10
		(c) Consider use of a flow diagram	20
Descriptive data	14*	(a) Give characteristics of study participants (eg demographic, clinical, social) and information on exposures and potential confounders	22-23
		(b) Indicate number of participants with missing data for each variable of interest	22-23
Outcome data	15*	Report numbers of outcome events or summary measures	22-23

For peer review only

Main results	16	(*a*) Give unadjusted estimates and, if applicable, confounder-adjusted estimates and their precision (eg, 95% confidence interval). Make clear which confounders were adjusted for and why they were included	N/A
		(*b*) Report category boundaries when continuous variables were categorized	24-25
		(*c*) If relevant, consider translating estimates of relative risk into absolute risk for a meaningful time period	N/A
Other analyses	17	Report other analyses done—eg analyses of subgroups and interactions, and sensitivity analyses	N/A
**Discussion**			
Key results	18	Summarise key results with reference to study objectives	12
Limitations	19	Discuss limitations of the study, taking into account sources of potential bias or imprecision. Discuss both direction and magnitude of any potential bias	12-13
Interpretation	20	Give a cautious overall interpretation of results considering objectives, limitations, multiplicity of analyses, results from similar studies, and other relevant evidence	12-13
Generalisability	21	Discuss the generalisability (external validity) of the study results	12-13
**Other information**			
Funding	22	Give the source of funding and the role of the funders for the present study and, if applicable, for the original study on which the present article is based	14

*Give information separately for exposed and unexposed groups.

Note: An Explanation and Elaboration article discusses each checklist item and gives methodological background and published examples of transparent reporting. The STROBE checklist is best used in conjunction with this article (freely available on the Web sites of PLoS Medicine at http://www.plosmedicine.org/, Annals of Internal Medicine at http://www.annals.org/, and Epidemiology at http://www.epidem.com/). Information on the STROBE Initiative is available at www.strobe-statement.org.

For peer review only

BMJ Open

Can we collect health-related quality of life information from anticoagulated atrial fibrillation participants who have recently experienced a bleed? An observational feasibility study in primary, and secondary care and through an online forum

Journal:	*BMJ Open*
Manuscript ID	bmjopen-2023-075335.R1
Article Type:	Original research
Date Submitted by the Author:	17-Jul-2023

Complete List of Authors:	Hutchings, Hayley; Swansea University, Swansea University Medical School<br>Lanyon, Kirsty; Swansea University, Swansea University Medical School<br>Holland, Gail; Swansea University, Swansea University Medical School<br>Alikhan, Raza; University Hospital of Wales, Department of Haematology<br>Jenkins, Rhys; Swansea City GP Cluster<br>LAING, HAMISH; Swansea University, VBHC Academy, School of Management<br>Hughes, Arfon; Swansea University<br>Lobban, Trudie; Arrythmia Alliance<br>Pollock, Kevin; Bristol-Myers Squibb Pharmaceuticals Ltd<br>Tod, Daniel; Swansea University, Swansea University Medical School<br>Lister, Steven; Bristol-Myers Squibb Pharmaceuticals Ltd

**Primary Subject Heading**:	Cardiovascular medicine
Secondary Subject Heading:	Haematology (incl blood transfusion), Health services research, Patient-centred medicine, Research methods
Keywords:	Feasibility Studies, Bleeding disorders & coagulopathies < HAEMATOLOGY, Anticoagulation < HAEMATOLOGY, Quality of Life

SCHOLARONE™
Manuscripts

I, the Submitting Author has the right to grant and does grant on behalf of all authors of the Work (as defined in the below author licence), an exclusive licence and/or a non-exclusive licence for contributions from authors who are: i) UK Crown employees; ii) where BMJ has agreed a CC-BY licence shall apply, and/or iii) in accordance with the terms applicable for US Federal Government officers or employees acting as part of their official duties; on a worldwide, perpetual, irrevocable, royalty-free basis to BMJ Publishing Group Ltd ("BMJ") its licensees and where the relevant Journal is co-owned by BMJ to the co-owners of the Journal, to publish the Work in this journal and any other BMJ products and to exploit all rights, as set out in our licence.

The Submitting Author accepts and understands that any supply made under these terms is made by BMJ to the Submitting Author unless you are acting as an employee on behalf of your employer or a postgraduate student of an affiliated institution which is paying any applicable article publishing charge ("APC") for Open Access articles. Where the Submitting Author wishes to make the Work available on an Open Access basis (and intends to pay the relevant APC), the terms of reuse of such Open Access shall be governed by a Creative Commons licence – details of these licences and which Creative Commons licence will apply to this Work are set out in our licence referred to above.

Other than as permitted in any relevant BMJ Author's Self Archiving Policies, I confirm this Work has not been accepted for publication elsewhere, is not being considered for publication elsewhere and does not duplicate material already published. I confirm all authors consent to publication of this Work and authorise the granting of this licence.

For peer review only

Can we collect health-related quality of life information from anticoagulated atrial fibrillation participants who have recently experienced a bleed? An observational feasibility study in primary, and secondary care and through an online forum

Hayley A Hutchings[1*], Kirsty Lanyon[1], Gail Holland[1], Raza Alikhan[2], Rhys Jenkins[3], Hamish Laing[4], Arfon Hughes[5], Trudie Lobban[6], Kevin G Pollock[7], Daniel Tod[1], Steven Lister[7]

1 Swansea University Medical School, Swansea University, Singleton Park, Swansea SA2 8PP, UK

*Corresponding author- Hayley A Hutchings

Email: h.a.hutchings@swansea.ac.uk

ORCID ID: https://orcid.org/0000-0003-4155-1741

2 Department of Haematology, University Hospital of Wales, Cardiff and Vale University Health Board, UK

3 Swansea City GP Cluster, Swansea, UK

4 VBHC Academy, School of Management, Swansea University

5 Public and Patient Involvement Representative

6 Arrhythmia Alliance, Public and Patient Representative, UK

7 Bristol Myers Squibb, Uxbridge Business Park, Sanderson Road, Uxbridge, Middlesex UB8 1DH, UK

Running head: health-related quality of life after a bleed

For peer review only - http://bmjopen.bmj.com/site/about/guidelines.xhtml

For peer review only

ABSTRACT

Objective

To evaluate the feasibility of recruiting participants diagnosed with atrial fibrillation (AF) taking oral anticoagulation therapies (OACs) and recently experiencing a bleed to collect health-related quality of life (HRQoL) information.

Design

Observational feasibility study. The study aimed to determine the feasibility of recruiting participants with minor and major bleeds, the most appropriate route for recruitment and the appropriateness of the Patient Reported Outcome Measures (PROMs) selected for collecting HRQoL information in AF patients, and the preferred format of the surveys.

Setting

Primary care, secondary care, and via an online patient forum.

Participants

The study population was adult patients (≥ 18) with Atrial Fibrillation (AF) taking oral anticoagulation therapies (OATs) who had experienced a recent major or minor bleed within the last four weeks.

Primary and Secondary outcome measures

Primary outcome:

Patient reported outcome measures (PROMs): EuroQol 5 dimensions-5 levels (EQ-5D-5L); Perception of anticoagulant treatment questionnaire, part 2 only (PACT-Q, part 2); Atrial fibrillation effect on quality of life (AFEQT)

Secondary outcomes:

Location of bleed; bleed severity; current treatment; patient perceptions of HRQoL in relation to bleeding events.

Results

We received initial expressions of interest from 103 participants. We subsequently recruited 32 participants to the study- 14 from primary care and 18 through the AF forum. No participants were recruited through secondary care. Despite 32 participants consenting, only 26 initial surveys were completed. We received follow-up surveys from 11 participants (8 primary care and 3 AF forum). COVID-19 had a major impact on the study.

Conclusions

Primary care was the most successful route for recruitment. Most participants recruited to the study experienced a minor bleed. Further ways to recruit in secondary care should be explored, especially to capture more serious bleeds.

Registration

For peer review only - http://bmjopen.bmj.com/site/about/guidelines.xhtml

The study was adopted onto the NIHR Portfolio (I.D. #47771) and registered with www.ClinicalTrials.gov (#NCT04921176) in February 2021.

Strengths and limitations of this study

- Multiple routes were used to identify potential participants with AF who had experienced a recent bleed- primary care, secondary care and through an online AF forum.
- Participants were able to complete the survey online (via REDCap®), on a paper-based form or over the telephone.
- Three validated patient reported outcome measures (PROMs) were used to gather different aspects of the impact of bleeding on AF patients.
- COVID- 19 impacted on the recruitment of participants and alternative mechanisms for recruitment need to be considered.

Key words: anticoagulation; bleeding disorders & coagulopathies; feasibility studies; health-related quality of life

Word count (excluding title page, abstract, references, figures, tables or acknowledgments)- 4072

For peer review only - http://bmjopen.bmj.com/site/about/guidelines.xhtml

INTRODUCTION

Atrial Fibrillation (AF) is the most common cardiac arrythmia (irregular heart rhythm disorder). Estimated overall prevalence has been reported to be between 0.4-1%[1 2]. UK estimates have suggested that AF prevalence is increasing and could be as high as 3.29%[3]. Many clinicians however debate the true global prevalence, believing that it is likely to be underestimated as many individuals are asymptomatic and are likely to go undiagnosed[4]. The rate increases with age over 60 years[5] and it is estimated to be much higher in individuals over 80 and in Western societies, with suggested rates as high as 14%[6]. There are known to be high numbers of individuals with undiagnosed AF[7-9].

AF is a chronic condition associated with significant risks of thromboembolism, stroke, and mortality[10 11]. Oral anticoagulation therapies (OATs)[1] are normally prescribed to reduce the risks of thromboembolism and AF-related stroke, but are associated with the side effect of bleeding, which in some cases can be serious[10 12 13]. These bleeding complications can include intracranial haemorrhage[14], gastrointestinal bleeding, and haematuria[15]. Bleeding as a side effect of OATs[16] remains a major challenge for clinicians that can often result in non-adherence and/or resistance to therapy[17]. While hospitalized AF-related stroke rates have declined and are significantly associated with increased anticoagulant uptake[18], the increased use of OATs has led to an increase in the number of bleeding events over time[10].

Patients are treated by healthcare providers with the primary goal of improving their health and well-being. Historically this improvement in health has been judged by improvement in biochemical, histological, radiological or clinical assessments. It is hypothesised that this approach does not always correlate with improvement from the patient perspective[19]. From a patient perspective, improving health is reflected in the documentation of their symptoms and experience of healthcare provision, which are more appropriately collected directly from the patient[20]. With a move towards shared-decision making and patient-centred care there is growing recognition within the healthcare community of the importance of the patient perspective and the need to consider patient-reported outcome measures (PROMs) as a key component of a holistic approach to patient care.

PROMs were initially developed for research use, and many regulatory authorities such as the European Medicines Agency (EMA) and the United States (US) Food and Drug Administration (FDA) advocate their use[21-23]. The U.S. Food and Drug Administration (FDA) defines a PROM as “any report of the status of a patient’s health condition that comes directly from the patient, without interpretation of the patient’s response by a clinician or anyone else”[22]. The European Medicines

For peer review only

For peer review only - http://bmjopen.bmj.com/site/about/guidelines.xhtml

Agency state that "Any outcome evaluated directly by the patient himself and based on the patient's perception of a disease and its treatment(s) is called a patient-reported outcome (PRO)"[24]. PROMs include quality of life (QoL) measures, disease severity scales, and patient experience measures.

The collection of PROMs aligns well with the increased drive within healthcare organisations for Value-Based healthcare, where organisations aim to achieve the best possible outcomes for patients with the available resources[25 26]. As more clinicians recognise the benefit of collecting PROMs in addition to measuring clinical outcomes, PROMs have seen an increased use in routine clinical practice[27].

AF is thought to have a detrimental effect on health-related quality of life (HRQoL) in patients [28-33]. This research has focused on exploring HRQoL following various treatments or procedures[34-36], the development of AF specific HRQoL measures[37-40], or the use of existing HRQoL measures in AF populations[41 42]. There is some evidence that bleeding events may have an adverse effect on patient HRQoL [43 44] but there has been limited exploration of HRQoL following a bleed in AF patients[42 45]. The aim of this study was to determine the feasibility of collecting HRQoL information from anticoagulated AF participants who had recently experienced a bleed. If this proves feasible, we plan to embark on a fully-powered study to evaluate the different dimensions of HRQoL of AF patients following a bleed.

METHODS

Selection of PROM to assess HRQoL

Prior to identifying sites and recruitment of participants, we undertook a process of PROM selection in order to select suitable tools that would allow us to assess HRQoL in AF participants who had experienced a recent bleed (reported elsewhere). This included a review of the literature to identify generic and disease specific PROMs that had been used in AF participants. Following the compilation of a list of possible PROMs, we undertook a rigorous evaluation of the identified PROMs that included an assessment of the overall validity of the PROMs, their content validity in terms of appropriateness of questions relating to bleeding events with AF, and how widely the PROM had previously been used in AF. This was informed by existing literature[46 47] and included a clear justification as to why the PROM was used[48]. In selecting the PROMs, we were also mindful to ensure the burden to participants was kept to a minimum, so also explored the number of questions within relevant PROMs. We worked with the two Public and Patient Involvement representatives when assessing the PROMs for help in considering their usefulness to the study.

Following the PROM scoping process, we agreed on three PROMs for use in the study:

- Euroquol 5 Dimensions-5 Levels (EQ-5D-5L)[49],

For peer review only - http://bmjopen.bmj.com/site/about/guidelines.xhtml

The EQ-5D is made up of the EQ-5D UK crosswalk scores which ranges between -0.594 to 1.000. A higher index score indicates a better QoL. The EQ-5D VAS (visual analogue scale) allows users to input a score of how good or bad their health is between 0 to 100, with a higher VAS score indicating a better QoL.

- Perception of Anticoagulant Treatment Questionnaire, Part 2 only (PACT-Q, Part 2)[50 51]; and

The PACT-Q Part 2 scores range from 0 to 100, for the convenience and satisfaction domains, with a higher score indicating higher convenience and higher satisfaction.

- Atrial fibrillation Effect on QualiTy-of-life (AFEQT)[41].

The disease specific AFEQT global and specific domain scores range from 0 to 100. A score of 0 corresponds to complete disability, while a score of 100 corresponds to no disability.

Participant Recruitment

We recruited adult participants (aged ≥18 years) who had a diagnosis of AF and were actively prescribed OATs for their AF. We identified participants who had experienced a recent bleed, up to a maximum of 30 days prior to enrolment. No upper age limit was imposed, but participants had to fit all other inclusion and exclusion criteria.

Inclusion criteria

- Adult participants (≥18 years)
- Participants who had a fluent understanding of English and were able to comprehend all study information and literature to provide fully informed consent
- Atrial fibrillation (AF) as the primary diagnosis
- Had a major or minor bleed up to a maximum of 30 days prior to date of enrolment
- Prescribed oral anticoagulation for AF

Exclusion Criteria

- Pregnant women
- Participants with active cancer
- Participants unable to consent for themselves
- Participants on concomitant antiplatelet therapy

As the study was designed to test feasibility for a fully powered definitive study, we aimed to recruit between 50 and 80 participants in the first instance based on recommended sample size guidance[52]. Figure 1 outlines the stages of the study.

Participating sites

For peer review only - http://bmjopen.bmj.com/site/about/guidelines.xhtml

For this feasibility study, we selected participants from the Swansea, UK area. We utilised three recruitment pathways for the study to test the feasibility of identifying and recruiting participants with minor and major bleeds:

- Primary care – Participants attending anticoagulation clinics of a large city General Practice cluster made up of 8 practices and serving almost 51,000 patients in Swansea, UK. Participants were identified by practice nurse/pharmacist as having a recent bleed.
- Secondary care – Participants admitted to the emergency department of a large UK University teaching hospital in Swansea, UK with a bleed were identified by research nurses/secondary care-based general practitioner.
- Direct participant – The study was publicised on the Arrhythmia Alliance website and newsletters (Arrhythmia Alliance UK (heartrhythmalliance.org) and individuals who formed part of this online forum and database were invited to participate.

When participants were identified through primary and secondary care, they were provided with a study information sheet for their consideration. If the patient was willing to participate, they were asked to return the expression of interest form to the study researcher in the supplied freepost envelope. The study researcher then contacted participants to provide them with a 'study pack'. As we aimed to determine the best approach for collecting the HRQoL information, we offered the participants the option of an electronic link to a REDCap® survey, a paper survey sent to their home with a freepost envelope, or the option to complete the survey by telephone. Individuals from the AF forum were initially provided with a weblink to the REDCap® survey through the website. Latterly, they were given the option to contact the researcher and be supplied with a paper survey.

For peer review only - http://bmjopen.bmj.com/site/about/guidelines.xhtml

Participants Survey Pack

We collected basic demographics from participants, details of their current OAT, existing co-morbidities, and details of any bleed(s) they had experienced (i.e. date, severity and bleed location). We asked all participants to complete three PROMs for the study survey at baseline: the Euroquol 5 Dimensions-5 Levels (EQ-5D-5L)[49], Perception of Anticoagulant Treatment Questionnaire (PACT-Q) Part 2[50 51], and the Atrial fibrillation Effect on QualiTy-of-life (AFEQT)[41].

We estimated that completion of all study questionnaires would take no longer than 30 minutes. Participants who agreed to participate were asked to complete the survey at two time points - once following consent, and then again at 90±14 days post-enrolment. Participants who completed the survey digitally via the REDCap® system received a second online link to complete the survey; those who completed paper forms were sent a follow up via post (along with a stamped addressed envelope for ease of return); and those who completed over the phone were contacted via telephone to complete the follow-up. Participants were contacted 3 weeks prior to the 90-day follow-up date. If no reply was received within 2 weeks of first correspondence, a reminder letter (with a second copy of all questionnaires) was sent, or a follow-up email or telephone call was made.

Data analysis

Quantitative data were checked, cleaned, and collated in REDCap® prior to being transferred to Stata Version 17.0.for analyses. As this was a feasibility study and not powered to detect differences between groups/OATs, no formal statistical comparisons were made. Data are presented descriptively as number, percentages, or means (standard deviation). HRQoL scores were calculated according to developers' guidance. Descriptive HRQoL scores are reported for AF participants following a minor or major bleed[53] while anticoagulated and by recruitment route. Categorisation of the bleed into minor or major was undertaken by a clinical professional (RA).

Ethics

We complied with Good Clinical Practice (GCP) guidelines and the General Data Protection Regulation (GDPR) in the conduct of the study and the collection of participant information. The study was given ethical approval from the London - Riverside Research Ethics Committee Research Ethics Committee (IRAS ID 279646), and Swansea Bay University Health Board R&D. The study research governance sponsor was Swansea University.

For peer review only

For peer review only - http://bmjopen.bmj.com/site/about/guidelines.xhtml

Participants were required to provide consent before enrolment into the study. Prior to consent we provided participants with an information sheet which ensured that they were clearly and fully informed about the purpose of the study, potential risks, use of their data, and their rights and responsibilities when participating in the study. They were also told that they had the right to withdraw at any point in the study. Participants who completed the survey via the REDCap® system consented to the study electronically. Participants completing paper forms were required to complete a paper consent and we obtained verbal consent for those completing over the telephone. We asked participants for informed consent for their anonymised data and interview quotes (where relevant) to be used in any future publications.

We were aware that participants may have experienced low mood or anxiety associated with their AF. If a participant reported, or showed signs of low mood, distress or anxiety, we encouraged them to discuss this with their primary or secondary care researcher and the participant was signposted to local relevant services or advised to contact their GP.

We stored all data securely within the password protected REDCap® secure web application while the study was conducted, and subsequently anonymised for analysis purposes.

COVID implications/changes

This study was originally conceived and designed before the COVID-19 pandemic. Shortly after receiving ethical approval and prior to the start of recruitment, the country went into its first lockdown to prevent onward transmission of the virus. Initially, the study was paused as we knew that participant recruitment through primary and secondary care would be impacted. As time progressed, however, we decided to adapt the study to take account of the situation and the change in operating practices across sites. The initial protocol included processes for face-to-face recruitment in primary care (through routine anticoagulation clinics) and in secondary care (through identification of AF bleeding cases through medical records and approach of patients on the wards).

To minimise any potential COVID-19 risk to both research staff and patients, it was agreed therefore that the study design should operate in line with current guidelines set out by the UK and Welsh Government for COVID-19 management. Up-to-date Government guidance was therefore followed throughout the study. This had a direct consequence on some aspects of study conduct. Study visits were therefore changed to ensure that they could be conducted remotely, until such time where face-to-face visits were permitted. Key members of the study team monitored relevant guidance for GP

For peer review only - http://bmjopen.bmj.com/site/about/guidelines.xhtml

surgeries and hospitals closely and necessary adjustments to study conduct were made accordingly, and consistently with any new information and/or restrictions as it was released. Where study tasks were conducted remotely, Health Research Authority (HRA) advice released for conducting research during the COVID-19 pandemic was followed, where applicable. Any study visits conducted in person took place with all required precautions in place to help prevent the spread of the virus.

RESULTS

We received initial expressions of interest from 85 participants in primary care. These expressions of interest were however not translated into participation in most cases. 31 questionnaires were sent out following successful initial contact with prepaid addressed envelopes for return but no further contact with the participant could be made. In addition, 18 participants started the survey on the AF forum. A total of 32 participants consented to participate in the study - 14 from primary care and 18 through the AF forum. No participants were recruited through secondary care.

COVID-19 had a major impact on the study, particularly in secondary care. We had difficulty recruiting in secondary care for three reasons. Firstly, many research nurses were transferred back to help manage frontline NHS activities; secondly, those who were still active had difficulty gaining access to participants and wards during lockdown; and thirdly, Welsh Government mandated that research nurse activity should be prioritised to COVID-19 vaccine studies. As a result, we focused on the two other recruitment routes, primary care and the AF forum.

Despite 32 participants consenting to participate, only 26 initial surveys were completed (14 from primary care and 12 from the AF forum), with 6 from the AF forum being abandoned prior to completion of any questions on REDCap®. We contacted primary care contacts at least twice if we had received an expression of interest from them.

We received follow-up surveys from 11 participants (8 primary care and 3 AF forum). Figure 2 illustrates the numbers of participants expressing an interest and subsequently consenting to participate in the study.

Table 1 illustrates the responses to the general questions from the 26 participants in the primary care and AF forum groups. We only had demographic information for the primary care group, where clinical data were cross referenced to patient records. The primary care patients were all white and

For peer review only - http://bmjopen.bmj.com/site/about/guidelines.xhtml

mostly female (79%). The HASBLED score (all less than 3) indicated that participants were at low risk of bleeding.

In both the primary care and AF forum groups, participants reported many pre-existing conditions. The most common were hypertension (reported by 43% of participants) and other (64%). There were a range of times since the bleed, including beyond 30 days, despite recruitment aiming to focus on bleeds of 30 days or less. Minor bleeding was more common than major bleeding in both groups, with 6/14 (42.9%) of participants in the primary care group and 5/12 (41.7%) in the AF forum group indicating that they had fairly frequent minor bleeds (1-2 per week or 1-2 per month). This was in contrast to the frequency of major bleeds, where 10/14 (71.4%) of participants from primary care and 7/12 (58.3%) from the AF forum indicated that they almost never had major bleeds. Clinical classification of the most recent bleed (where documented) indicated that there were 18 minor, 2 major and 1 unknown.

Table 2 illustrates the HRQoL scores from the three questionnaires. The mean EQ-5D UK crosswalk and EQ-5D VAS scores in the primary care group were lower than that in the AF forum group at baseline (0.470 vs 0.809 crosswalk; 58.9 vs 70.6 VAS). The follow up scores for both the cross walk and VAS scores in the primary care group were higher, but still less than the AF forum group (0.694 vs 0.893 crosswalk; 60.1 vs 83.0 VAS).

The AFEQT overall and subscale scores were largely in the positive range indicating limited disability due to AF. At baseline, the global AFEQT score, symptom, daily activities, and treatment concern domain scores in the primary care and AF forum group were similar for the global, symptom and daily activities scores (64.8 vs 65.7 global; 76.4 vs 78.0 symptom; 50.0 vs 58.7 daily activities). The participants in the primary care group had less treatment concern at baseline that the AF forum group (76.9 vs 66.9; treatment concern). The global and symptom scores showed little change between the baseline and follow-up survey in the primary care group (64.8 vs 63.3 global; 76.4 vs 74.0). The primary care group score did however improve for daily activities (50.0 vs 59.8; daily activities) and deteriorate for treatment concern (76.9 vs 59.1). In the AF forum group, there was an improvement in the global score (65.7 vs 77.2 global) and little change in the symptom score (78.0 vs 81.9 symptom). There was also an improvement in the daily activities (58.7 vs 76.4 daily activities) and treatment concern (66.9 vs 75.0) score in the AF group between baseline and follow-up. Both groups had a deterioration in response to the two AFEQT satisfaction questions (Q19 and 20, see Table 2). These questions relate

For peer review only - http://bmjopen.bmj.com/site/about/guidelines.xhtml

to how well they think their current treatment controls their AF and the extent to which their treatment has relieved their symptoms.

The PACT-Q2 convenience scores were comparable between the primary care and AF forum groups at baseline (87.0 vs 86.5 convenience). There was little change in these scores at follow-up in the primary care (87.0 vs 86.8) or the AF forum group (86.5 vs 86.5). The initial PACT-Q2 satisfaction scores were again comparable between the primary care and AF forum group (65.4 vs 67.5 satisfaction). The primary care group however showed a deterioration in the satisfaction score at follow-up (65.4 vs 61.6), whereas the AF forum group showed an improvement in scores (67.5 vs 76.2) at follow-up.

Due to the small numbers participating in the surveys, it is difficult to infer any meaningful conclusions from these results and as such they should be interpreted with caution.

DISCUSSION

After receiving the necessary ethics and R&D approvals for the study, the UK went into lockdown, and we were forced to initially pause the study. When we were in a position to restart, we had to modify the study to take account of changing healthcare practices. This included remote engagement and varying formats for completion of the questionnaires. We used various approaches to increase recruitment, including the option to complete the survey on paper, through an online survey link or over the phone. Despite receiving many expressions of interest and making multiple phone calls, we were unable to make contact with most patients who had expressed an interest in participating. The study was open for a year longer than planned (2 years in total) after which the research management group made the decision to close.

Recruitment in secondary care was mostly impacted and we were unable to recruit any patients through this route. This was largely due to nurses being reassigned to front-line roles, limited access to wards, and a focus on recruitment to Covid-19 studies. We were still able to recruit through primary care and the AF online forum. There were however a number of incomplete questionnaires submitted through the online forum. This may indicate that patients find online forms difficult to complete[54] or that they were unsure of the relevance of the questions or found the survey too long. Separate qualitative interviews with patients will explore these concepts further.

For peer review only - http://bmjopen.bmj.com/site/about/guidelines.xhtml

Primary care therefore appeared to be the best way to recruit participants. This may have been because they were introduced to the study by the primary care pharmacist whom they knew, and they were therefore not contacted 'cold'. We received initial expressions of interest in primary care from 90 participants. This did not however translate into 90 completed questionnaires. This may be because of the time lapse between discussing the study and subsequently being contacted by the researcher or misunderstanding regarding the rationale for the study. Had the participants received the questionnaire on initial contact, this may have increased the completion rate.

Most of the patients that completed the survey had minor bleeds. This may be due to the fact that most patients were recruited through primary care. Had we recruited through secondary care, we may have seen more major bleeds.

Selecting the PROMs for the study was challenging. The focus of the study was to test the feasibility of collecting data regarding HRQoL in AF participants on OATs who experienced a bleed. Details of the selection process for the PROMs will be reported separately, but the final decision was based on 1) previous use of the PROMs with the AF population; 2) assessment of the validity of the PROMs; and 3) relevance of the questions to a bleeding event. There were few PROMs that had questions that specifically focused on the bleed. This may have also contributed to the fact that the response rate was lower than expected.

It was difficult to interpret the HRQoL scores as the study was not powered to detect any statistically significant differences. In addition, there was a great deal of variability in the scores across the different scales even within the same groups. Due to the limited number of participants recruited and the variability in the scores obtained, contextualising these scores with existing literature is therefore difficult. Further studies should explore whether the type of OAT has any impact on HRQoL, particularly as some OATs are less likely to cause bleeding. We collected information regarding existing co-morbidities and a future study should also explore whether any of these conditions are likely to affect the risk of bleeding.

Further work needs to be undertaken to explore alternative ways of recruiting through secondary care. In our study we utilised research nurses with the aim of accessing ward admission records to identify potential patients. This mechanism for recruitment was particularly impacted by the pandemic; access to the wards was restricted, many research nurses were moved back to frontline activities, and covid-19 research studies were prioritised. Recruitment through secondary care may

For peer review only - http://bmjopen.bmj.com/site/about/guidelines.xhtml

For peer review only

be improved by working directly with clinicians who manage AF patients on OATs. It is likely that this would also increase the number of participants recruited with major bleeds and help to determine the most appropriate time to approach participants, especially as they may be too ill during their inpatient episode.

The way we recruited through primary care could also be streamlined, with participants being invited to participate following their initial contact with the lead clinician managing the bleeding clinics rather than being contacted by the researcher following completion of an expression of interest. The relevance of the questionnaires may have impacted on the completion of the survey through the online forum, so use of a more specific PROM that focused on bleeding issues may facilitate this.

Despite the challenges of undertaking the study in the pandemic, we were still able to recruit 32 patients. A further fully- powered study is now needed to explore whether HRQoL is impacted by bleeding episodes in AF patients on OATs, and whether the extent of the bleed has an effect on HRQoL.

AUTHOR CONTRIBUTIONS

HAH and SL conceived the study. HAH designed the initial study proposal, led on interpretation of the findings and drafted the manuscript. KL was responsible for site set-up, ethical approvals, participant recruitment and data collection; participated in interpretation of findings and provided input on the manuscript. GH provided overall study governance support, specifically in relation to ethical aspects, provided input into final protocol, participated in interpretation of findings and provided input on the manuscript. RA provided input into final protocol, was responsible for clinical interpretation of bleeds, participated in interpretation of findings and provided input on the manuscript. RJ led primary care recruitment, provided input into final protocol, participated in interpretation of findings and provided input on the manuscript. TL led AF forum recruitment, provided input into final protocol, provided PPI input on survey design, participated in interpretation of findings and was provided input on the manuscript. AH provided input into final protocol, provided PPI input on survey design, participated in interpretation of findings and provided input on the manuscript. DT was responsible for writing the analysis plan, final analysis and interpretation of the findings and provided input on the manuscript. HL, KP provided input into final protocol, participated in interpretation of findings and provided input on the manuscript. All authors reviewed and approved the final manuscript.

ACKNOWLEDGEMENTS

For peer review only

For peer review only - http://bmjopen.bmj.com/site/about/guidelines.xhtml

We would like to thank all participants for completing the survey, the general practice nurses from Swansea Bay City Cluster who helped with recruitment, and the Arrhythmia Alliance for hosting the online survey.

FUNDING AND GOVERNANCE

The Chief Investigator of the study was Professor Hayley Hutchings, Swansea Trials Unit, Swansea University (SU). Under the UK Policy Framework for Health and Social Care Research SU met the requirements of research governance sponsor. BMS/Pfizer funded the research (grant number: CV185-770) and under the UK policy framework were part of a partnership that accepted overall responsibility for proportionate and effective arrangements to be in place to set up, run and report the research project. They were represented on the Trial Steering Committee throughout the project.

CONFLICT OF INTEREST

Hayley A Hutchings leads this independent research which was funded by Pfizer and Bristol Myers Squibb.

Hamish Laing is an employee of Swansea University who has received a grant for a Value-Based Healthcare programme of work from Pfizer Ltd., which he leads and support to attend a meeting.

Steven Lister was an employee of BMS at the time of the study and holds stock in BMS and Pfizer.

Kevin Pollock is an employee of BMS and holds stock in BMS.

Gail Holland, Kirsty Lanyon, Arfon Hughes, Rhys Jenkins, Trudie Lobban, declare that they have no conflict of interest.

DATA SHARING AGREEMENT

The anonymised dataset are available on the Zenodo open access data repository[55].

For peer review only - http://bmjopen.bmj.com/site/about/guidelines.xhtml

REFERENCES

1. Liu GJ, Wang YF, Chen PY, et al. The efficacy and safety of novel oral anticoagulants for the preventive treatment in atrial fibrillation patients: a systematic review and meta-analysis. *Drug Deliv* 2014;21(6):436-52. doi: 10.3109/10717544.2013.873500 [published Online First: 20140108]

2. Zulkifly H, Lip GYH, Lane DA. Epidemiology of atrial fibrillation. *Int J Clin Pract* 2018;72(3):e13070. doi: 10.1111/ijcp.13070 [published Online First: 2018/03/02]

3. Adderley NJ, Ryan R, Nirantharakumar K, et al. Prevalence and treatment of atrial fibrillation in UK general practice from 2000 to 2016. *Heart* 2019;105(1):27-33. doi: 10.1136/heartjnl-2018-312977 [published Online First: 2018/07/12]

4. Patel NJ, Atti V, Mitrani RD, et al. Global rising trends of atrial fibrillation: a major public health concern. *Heart* 2018;104(24):1989-90. doi: 10.1136/heartjnl-2018-313350 [published Online First: 2018/06/17]

5. Darby-Stewart A, Dachs R, Graber MA. Rivaroxaban vs. warfarin for stroke prevention in patients with nonvalvular atrial fibrillation. *Am Fam Physician* 2012;85(6):577-86.

6. Kodani E, Atarashi H. Prevalence of atrial fibrillation in Asia and the world. *Journal of Arrhythmia* 2012;28(6):330-37.

7. Turakhia MP, Shafrin J, Bognar K, et al. Estimated prevalence of undiagnosed atrial fibrillation in the United States. *PLoS One* 2018;13(4):e0195088. doi: 10.1371/journal.pone.0195088 [published Online First: 2018/04/13]

8. Clua-Espuny JL, Lechuga-Duran I, Bosch-Princep R, et al. Prevalence of undiagnosed atrial fibrillation and of that not being treated with anticoagulant drugs: the AFABE study. *Rev Esp Cardiol (Engl Ed)* 2013;66(7):545-52. doi: 10.1016/j.rec.2013.03.003 [published Online First: 2014/04/30]

9. Suissa L, Lachaud S, Mahagne MH. Optimal timing and duration of continuous electrocardiographic monitoring for detecting atrial fibrillation in stroke patients. *J Stroke*

For peer review only

For peer review only - http://bmjopen.bmj.com/site/about/guidelines.xhtml

Cerebrovasc Dis 2013;22(7):991-5. doi: 10.1016/j.jstrokecerebrovasdis.2012.01.015 [published Online First: 2012/02/22]

10. Hansen PW, Sehested TSG, Fosbol EL, et al. Trends in warfarin use and its associations with thromboembolic and bleeding rates in a population with atrial fibrillation between 1996 and 2011. *PLoS One* 2018;13(3):e0194295. doi: 10.1371/journal.pone.0194295 [published Online First: 20180316]
11. Kannel WB, Wolf PA, Benjamin EJ, et al. Prevalence, incidence, prognosis, and predisposing conditions for atrial fibrillation: population-based estimates. *Am J Cardiol* 1998;82(8A):2N-9N. doi: 10.1016/s0002-9149(98)00583-9
12. Turagam MK, Parikh V, Afzal MR, et al. Replacing warfarin with a novel oral anticoagulant: Risk of recurrent bleeding and stroke in patients with warfarin ineligible or failure in patients with atrial fibrillation (The ROAR study). *J Cardiovasc Electrophysiol* 2017;28(8):853-61. doi: 10.1111/jce.13254 [published Online First: 20170608]
13. Lamberts M, Staerk L, Olesen JB, et al. Major Bleeding Complications and Persistence With Oral Anticoagulation in Non-Valvular Atrial Fibrillation: Contemporary Findings in Real-Life Danish Patients. *J Am Heart Assoc* 2017;6(2) doi: 10.1161/JAHA.116.004517 [published Online First: 20170214]
14. Hankey GJ. Intracranial hemorrhage and novel anticoagulants for atrial fibrillation: what have we learned? *Curr Cardiol Rep* 2014;16(5):480. doi: 10.1007/s11886-014-0480-9
15. Wallis CJD, Juvet T, Lee Y, et al. Association Between Use of Antithrombotic Medication and Hematuria-Related Complications. *JAMA* 2017;318(13):1260-71. doi: 10.1001/jama.2017.13890
16. Khan F, Huang H, Datta YH. Direct oral anticoagulant use and the incidence of bleeding in the very elderly with atrial fibrillation. *J Thromb Thrombolysis* 2016;42(4):573-8. doi: 10.1007/s11239-016-1410-z

For peer review only

17. Dar T, Yarlagadda B, Vacek J, et al. Management of Stroke risk in atrial fibrillation patients with bleeding on Oral Anticoagulation Therapy-Role of Left Atrial Appendage Closure, Octreotide and more. *J Atr Fibrillation* 2017;10(4):1729. doi: 10.4022/jafib.1729

18. Cowan JC, Wu J, Hall M, et al. A 10 year study of hospitalized atrial fibrillation-related stroke in England and its association with uptake of oral anticoagulation. *Eur Heart J* 2018;39(32):2975-83. doi: 10.1093/eurheartj/ehy411 [published Online First: 2018/07/10]

19. Pakhomov SV, Jacobsen SJ, Chute CG, et al. Agreement between patient-reported symptoms and their documentation in the medical record. *Am J Manag Care* 2008;14(8):530-9.

20. Hutchings HA, Alrubaiy L. Patient-Reported Outcome Measures in Routine Clinical Care: The PROMise of a Better Future? *Dig Dis Sci* 2017;62(8):1841-43. doi: 10.1007/s10620-017-4658-z

21. European Medicines Agency. Reflection paper on th regulatory guidance for the use of Health-Related Quality of Life (HRQL) measures in the evaluation of medicinal products. Available from: https://www.ema.europa.eu/en/documents/scientific-guideline/reflection-paper-regulatory-guidance-use-healthrelated-quality-life-hrql-measures-evaluation_en.pdf 2005 [accessed 5 November 2019.

22. U.S. Department of Health and Human Services, Food and Drug Administration, Center for Drug Evaluation and Research (CDER), et al. Guidance for Industry Patient-Reported Outcome Measures: Use in Medical Product Development to Support Labeling Claims. Available from: https://www.fda.gov/downloads/Drugs/GuidanceComplianceRegulatoryInformation/Guidances/UCM193282.pdf 2009 [accessed 5 November 2019.

23. Revicki DA, Regulatory I, Patient-Reported Outcomes Task Force for the International Society for Quality of Life R. FDA draft guidance and health-outcomes research. *Lancet* 2007;369(9561):540-2. doi: 10.1016/S0140-6736(07)60250-5

For peer review only - http://bmjopen.bmj.com/site/about/guidelines.xhtml

24. Downing J, Namisango E, Harding R. Outcome measurement in paediatric palliative care: lessons from the past and future developments. *Ann Palliat Med* 2018;7(Suppl 3):S151-S63. doi: 10.21037/apm.2018.04.02 [published Online First: 2018/09/06]

25. Porter ME, Larsson S, Lee TH. Standardizing Patient Outcomes Measurement. *N Engl J Med* 2016;374(6):504-6. doi: 10.1056/NEJMp1511701

26. Porter ME, Lee TH. The Strategy That Will Fix Health Care. *Harvard Bus Rev* 2013;91(12):24-24.

27. Black N. Patient reported outcome measures could help transform healthcare. *BMJ* 2013;346:f167. doi: 10.1136/bmj.f167

28. Peinado R, Arribas F, Ormaetxe JM, et al. Variation in quality of life with type of atrial fibrillation. *Rev Esp Cardiol* 2010;63(12):1402-9. doi: 10.1016/s1885-5857(10)70274-7 [published Online First: 2010/12/15]

29. Zhang L, Gallagher R, Neubeck L. Health-related quality of life in atrial fibrillation patients over 65 years: A review. *Eur J Prev Cardiol* 2015;22(8):987-1002. doi: 10.1177/2047487314538855 [published Online First: 2014/06/14]

30. Deaton C, Dunbar SB, Moloney M, et al. Patient experiences with atrial fibrillation and treatment with implantable atrial defibrillation therapy. *Heart Lung* 2003;32(5):291-9. doi: 10.1016/s0147-9563(03)00074-8 [published Online First: 2003/10/07]

31. Aliot E, Botto GL, Crijns HJ, et al. Quality of life in patients with atrial fibrillation: how to assess it and how to improve it. *Europace* 2014;16(6):787-96. doi: 10.1093/europace/eut369 [published Online First: 2014/01/29]

32. Ong L, Cribbie R, Harris L, et al. Psychological correlates of quality of life in atrial fibrillation. *Qual Life Res* 2006;15(8):1323-33. doi: 10.1007/s11136-006-0029-5 [published Online First: 20060707]

33. Thrall G, Lane D, Carroll D, et al. Quality of life in patients with atrial fibrillation: a systematic review. *Am J Med* 2006;119(5):448 e1-19. doi: 10.1016/j.amjmed.2005.10.057

For peer review only

For peer review only - http://bmjopen.bmj.com/site/about/guidelines.xhtml

34. Pokorney SD, Kim S, Thomas L, et al. Cardioversion and subsequent quality of life and natural history of atrial fibrillation. *Am Heart J* 2017;185:59-66. doi: 10.1016/j.ahj.2016.10.018 [published Online First: 2017/03/08]

35. Sun Y, Zhu J, Ma C, et al. Stroke Risk Status, Anticoagulation Treatment, and Quality-of-Life in Chinese Patients with Atrial Fibrillation: China Registry of Atrial Fibrillation (CRAF). *Cardiovasc Ther* 2019;2019:7372129. doi: 10.1155/2019/7372129 [published Online First: 2019/11/28]

36. Bai Y, Bai R, Wu JH, et al. Differences in Quality of Life Between Atrial Fibrillation Patients with Low Stroke Risk Treated With and Without Catheter Ablation. *J Am Heart Assoc* 2015;4(9):e002130. doi: 10.1161/JAHA.115.002130 [published Online First: 2015/09/18]

37. Coyne KS, Edvardsson N, Ryden A. Development and Validation of the AFImpact: An Atrial Fibrillation-Specific Measure of Patient-Reported Health-Related Quality of Life. *Value Health* 2017;20(10):1355-61. doi: 10.1016/j.jval.2017.06.005 [published Online First: 2017/12/16]

38. Badia X, Arribas F, Ormaetxe JM, et al. Development of a questionnaire to measure health-related quality of life (HRQoL) in patients with atrial fibrillation (AF-QoL). *Health Qual Life Outcomes* 2007;5:37. doi: 10.1186/1477-7525-5-37 [published Online First: 2007/07/06]

39. Arribas F, Ormaetxe JM, Peinado R, et al. Validation of the AF-QoL, a disease-specific quality of life questionnaire for patients with atrial fibrillation. *Europace* 2010;12(3):364-70. doi: 10.1093/europace/eup421 [published Online First: 2010/01/09]

40. Medin J, Arbuckle R, Abetz L, et al. Development and validation of the AFSymp: an atrial fibrillation-specific measure of patient-reported symptoms. *Patient* 2014;7(3):319-27. doi: 10.1007/s40271-014-0058-z

41. Spertus J, Dorian P, Bubien R, et al. Development and validation of the Atrial Fibrillation Effect on QualiTy-of-Life (AFEQT) Questionnaire in patients with atrial fibrillation. *Circ Arrhythm*

For peer review only

For peer review only - http://bmjopen.bmj.com/site/about/guidelines.xhtml

Electrophysiol 2011;4(1):15-25. doi: 10.1161/CIRCEP.110.958033 [published Online First: 2010/12/17]

42. Wang K, Li H, Kwong WJ, et al. Impact of Spontaneous Extracranial Bleeding Events on Health State Utility in Patients with Atrial Fibrillation: Results from the ENGAGE AF-TIMI 48 Trial. *J Am Heart Assoc* 2017;6(8) doi: 10.1161/JAHA.117.006703 [published Online First: 2017/09/02]

43. Res LCS, Lubberts B, Shah SH, et al. Health-related quality of life after adverse bleeding events associated with antithrombotic drug therapy - A systematic review. *Hellenic J Cardiol* 2019;60(1):3-10. doi: 10.1016/j.hjc.2018.06.012

44. Radaideh KM, Matalqah LM. Health-related quality of life among atrial fibrillation patients using warfarin therapy. *Epidemiology Biostatistics and Public Health* 2018;15(1):e12763-1. doi: 10.2427/12763

45. De Caterina R, Bruggenjurgen B, Darius H, et al. Quality of life and patient satisfaction in patients with atrial fibrillation on stable vitamin K antagonist treatment or switched to a non-vitamin K antagonist oral anticoagulant during a 1-year follow-up: A PREFER in AF Registry substudy. *Arch Cardiovasc Dis* 2018;111(2):74-84. doi: 10.1016/j.acvd.2017.04.007 [published Online First: 2017/09/25]

46. Kotecha D, Ahmed A, Calvert M, et al. Patient-Reported Outcomes for Quality of Life Assessment in Atrial Fibrillation: A Systematic Review of Measurement Properties. *PLoS One* 2016;11(11):e0165790. doi: 10.1371/journal.pone.0165790 [published Online First: 20161101]

47. Reynolds MR, Ellis E, Zimetbaum P. Quality of life in atrial fibrillation: measurement tools and impact of interventions. *J Cardiovasc Electrophysiol* 2008;19(7):762-8. doi: 10.1111/j.1540-8167.2007.01091.x [published Online First: 20080205]

48. Vibeke Høgh V, Delmar C, Riahi S, et al. Quality of Life - Exemplified through Patients Living with Atrial Fibrillation. *J Nurs Care* 2014; 3(2) doi: http://dx.doi.org/10.4172/2167-1168.1000150

For peer review only - http://bmjopen.bmj.com/site/about/guidelines.xhtml

49. EQ-5D. EQ-5D-5L. About. Available from: https://euroqol.org/eq-5d-instruments/eq-5d-5l-about/ (Access date 13 January 2022) 2009 [

50. Prins MH, Guillemin I, Gilet H, et al. Scoring and psychometric validation of the Perception of Anticoagulant Treatment Questionnaire (PACT-Q). *Health Qual Life Outcomes* 2009;7:30. doi: 10.1186/1477-7525-7-30 [published Online First: 20090407]

51. Prins MH, Marrel A, Carita P, et al. Multinational development of a questionnaire assessing patient satisfaction with anticoagulant treatment: the 'Perception of Anticoagulant Treatment Questionnaire' (PACT-Q). *Health Qual Life Outcomes* 2009;7:9. doi: 10.1186/1477-7525-7-9 [published Online First: 20090206]

52. Lancaster GA, Dodd S, Williamson PR. Design and analysis of pilot studies: recommendations for good practice. *J Eval Clin Pract* 2004;10(2):307-12. doi: 10.1111/j.2002.384.doc.x

53. Schulman S, Kearon C, Subcommittee on Control of Anticoagulation of the S, et al. Definition of major bleeding in clinical investigations of antihemostatic medicinal products in non-surgical patients. *J Thromb Haemost* 2005;3(4):692-4. doi: 10.1111/j.1538-7836.2005.01204.x [published Online First: 2005/04/22]

54. Calvert MJ, Cruz Rivera S, Retzer A, et al. Patient reported outcome assessment must be inclusive and equitable. *Nat Med* 2022;28(6):1120-24. doi: 10.1038/s41591-022-01781-8

[dataset] 55. Hutchings HA, Lanyon K, Holland G, et al. Can we collect health-related quality of life information from anticoagulated atrial fibrillation participants who have recently experienced a bleed? An observational feasibility study in primary, and secondary care and through an online forum. Available from: https://zenodo.org/record/8147317. Zenodo, 2023.

For peer review only

For peer review only - http://bmjopen.bmj.com/site/about/guidelines.xhtml

FIGURES

Figure 1. Flow chart of the stages involved in the study.

Figure 2. Flow chart of the participants recruited to the study and subsequently analysed.

For peer review only

For peer review only - http://bmjopen.bmj.com/site/about/guidelines.xhtml

TABLES

For peer review only

For peer review only - http://bmjopen.bmj.com/site/about/guidelines.xhtml

Table 1. Demographics of the recruited participants

	Primary care (n=14)	AF forum (n=12)*
Ethnicity		
White	14(100%)	n/a
Sex (M:F)	3:11	n/a
CHA_2DS_2-VASc		
Mean (Min, Max)	3.64 (1, 6)	n/a
HAS-BLED		
0	1(7.1%)	n/a
1	5(35.7%)	
2	8(57.1%)	
Medication		
Apixaban	7(50.0%)	1(8.3%)
Dabigatran	0(0%)	1(8.3%)
Rivaroxaban	5(35.7%)	4(33.3%)
Warfarin	2(14.3%)	2(16.7%)
Xarelto	0(0%)	1(8.3%)
Missing	1(7.1%)	3(25.0%)
Co-morbidities/pre-existing disease		
Congestive heart failure	0(0%)	1(8.3%)
Hypertension	6(42.9%)	5(41.7%)
Hypotension	1(7.1%)	0(0%)
Ischaemic heart disease	0(0%)	1(8.3%)
Chronic Obstructive Pulmonary Disease	2(14.3%)	0(0%)
Diabetes related heart disease	1(7.1%)	1(8.3%)
Other	9(64.3%)	3(25.0%)
Days since bleed		
0-7	3(21.4%)	2(16.7%)
8-30	0(0%)	2(16.7)
31-60	1(7.1%)	4(33.3%)
60+	1(7.1%)	0(0%)
Unknown	9(64.3%)	4(33.3%)
Location of Bleed		
Internal	3(21.4%)	4(33.3%)
External	7(50%)	4((33.3%)
Unsure	1(7.1%)	1(8.3%)
Missing	3(21.4%)	3(25.0%)
Body location of external bleed		
Head	0(0%)	2(50.0%)
Neck	1(14.3%)	0(0%)
Forearm	2(28.6%)	0(0%)
Calf/shin	1(14.3%)	1(25.0%)
Feet, ankle, toes	1(14.3%)	0(0%)
Haemorrhoids	1(14.3%)	0(0%)
Missing	1(14.3%)	1(25.0%)
Body location of internal bleed		
Head	2(66.7%)	0 (0%)
Respiratory system	0(0%)	1(25.0%)

For peer review only

For peer review only - http://bmjopen.bmj.com/site/about/guidelines.xhtml

Reproductive system	0(0%)	1(25.0%)
Other	1(33.3%)	2(50.0%)
Frequency of minor bleeds		
1-2 times per week	4(28.6%)	1(8.3%)
1-2 times per month	2(14.3%)	4(33.3%)
1-2 times over 6 months	3(21.4%)	2(16.7%)
1-2 times a year	0(0%)	1(8.3%)
Almost never	3(21.4%)	2(16.7%)
Missing	2(14.3%)	2(16.7%)
Frequency of major bleeds		
1-2 per week	1(7.1%)	0(0%)
1-2 times a year	0(0%)	2(16.7%)
Almost never	10(71.4%)	7(58.3%)
Missing	3(21.4%)	3(25.0%)

- Demographic/symptom information was not collected on the AF forum as it could not be clinically verified.

For peer review only

For peer review only - http://bmjopen.bmj.com/site/about/guidelines.xhtml

Table 2. Health-related quality of life (HRQoL) scores at baseline and follow up split by primary care and AF forum patients measured using the EQ5D, AFEQT and PACT questionnaires.

a) EQ5D UK crosswalk scores

EQ-5D UK Crosswalk*						
Mean (Min, Max)	First Survey		Follow-up Survey		Total	
Primary Care	n=13	0.470 (-0.200, 1.000)	n=8	0.694 (0.393, 1.000)	n=21	0.555 (-0.200, 1.000)
Online Forum	n=12	0.809 (0.476, 1.000)	n=3	0.893 (0.679, 1.000)	n=15	0.826 (0.476, 1.000)
Total	n=25	0.633 (-0.200, 1.000)	n=11	0.748 (0.393, 1.000)	n=36	0.668 (-0.200, 1.000)

* The crosswalk has a score range of -0.594 – 1.000.

EQ-5D UK VAS						
Mean (Min, Max)	First Survey		Follow-up Survey		Total	
Primary Care	n=13	58.9 (22, 92)	n=8	60.1 (25, 98)	n=21	59.4 (22, 98)
Online Forum	n=12	70.6 (25, 92)	n=3	83.0 (70, 90)	n=15	73.1 (25, 92)
Total	n=25	64.5 (22, 92)	n=11	66.4 (25, 98)	n=36	65.1 (22, 98)

b) AFEQT Scores

AFEQT		
Mean (Min, Max)	First Survey	Follow-up Survey
Global Score		
Primary Care	64.8 (18.5, 98.1)	63.3 (37.0, 91.7)
AF Forum	65.7 (25.9, 94.4)	77.2 (50.5, 93.5)
Symptom Domain Score		
Primary Care	76.4 (33.3, 100)	74.0 (37.5, 95.8)
AF Forum	78.0 (37.5, 100)	81.9 (58.3, 95.8)
Daily Activities Domain Score		
Primary Care	50.0 (14.6, 100)	59.8 (8.3, 95.8)
AF Forum	58.7 (10.4, 91.7)	76.4 (47.9, 95.8)
Treatment Concern Domain Score		
Primary Care	76.9 (13.9, 100)	59.1 (11.1, 83.3)
AF Forum	66.9 (33.3, 91.4)	75.0 (50.0, 91.7)

AFEQT Satisfaction Questions		
Mean (Min, Max)	First Survey	Follow-up Survey
Q19. How well your current treatment controls your atrial fibrillation?		
Primary Care	75.0 (50.0, 100)	62.5 (16.7, 100)
AF Forum	65.3 (0.0, 83.3)	61.1 (50.0, 66.7)
Q20. The extent to which treatment has relieved your symptoms of atrial fibrillation?		

For peer review only - http://bmjopen.bmj.com/site/about/guidelines.xhtml

Primary Care	73.6 (50.0, 100)	62.5 (16.7, 83.3)
AF Forum	66.7 (0.0, 100)	61.1 (50.0, 66.7)

c) PACT-Q Part 2 Scores

PACT-Q Scores		
Mean (Min, Max)	First Survey	Follow-up Survey
Convenience score		
Primary Care	87.0 (59.6, 100)	86.8 (69.2, 100)
AF Forum	86.5 (65.4, 98.1)	86.5 (76.9, 98.1)
Satisfaction score		
Primary Care	65.4 (39.3, 96.4)	61.6 (50.0, 78.6)
AF Forum	67.5 (39.3, 89.3)	76.2 (64.3, 92.9)

For peer review only

For peer review only - http://bmjopen.bmj.com/site/about/guidelines.xhtml

Figure 1. Flow chart of the stages involved in the study

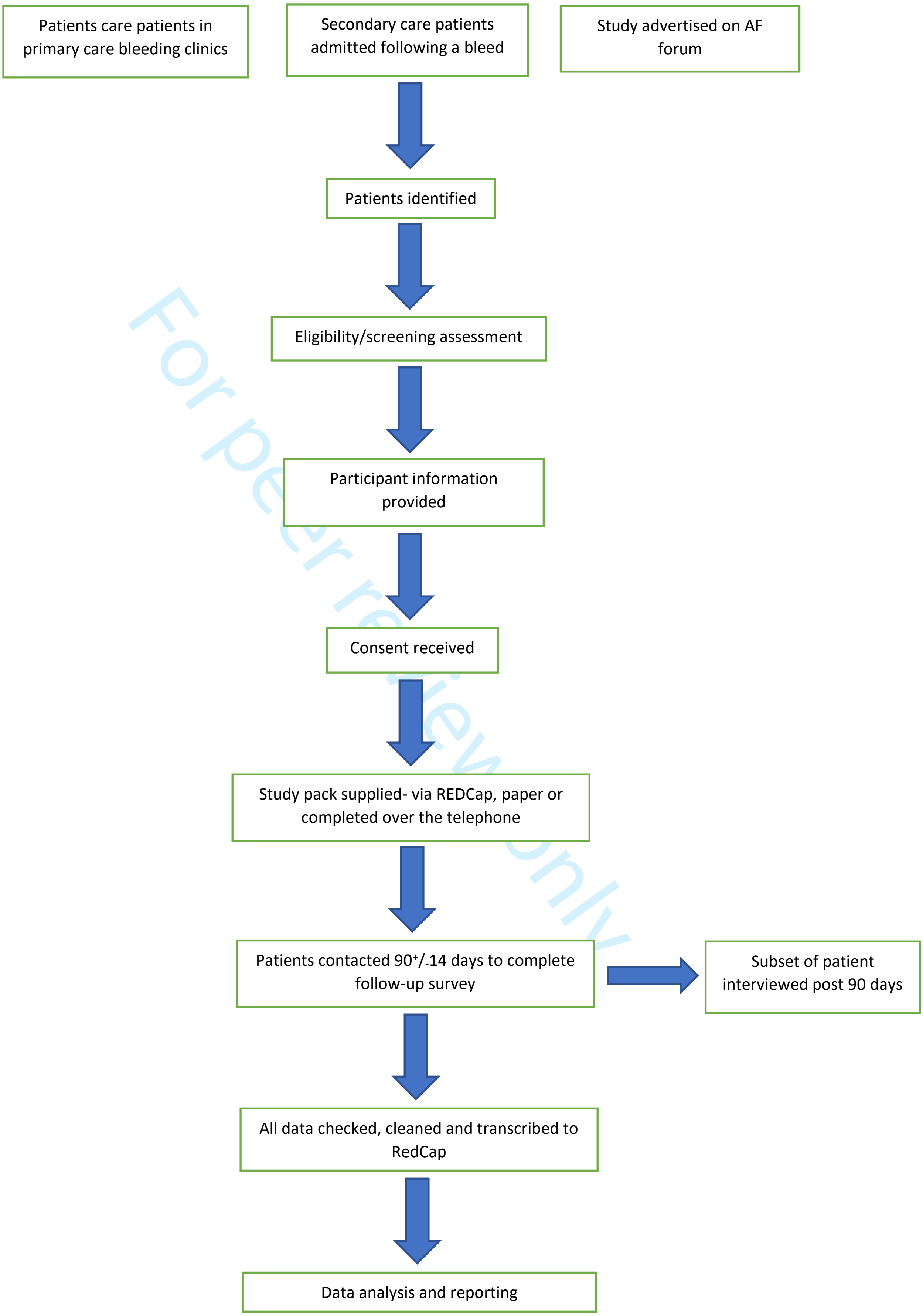

For peer review only

Figure 2. Flow chart of the participants recruited to the study and subsequently analysed

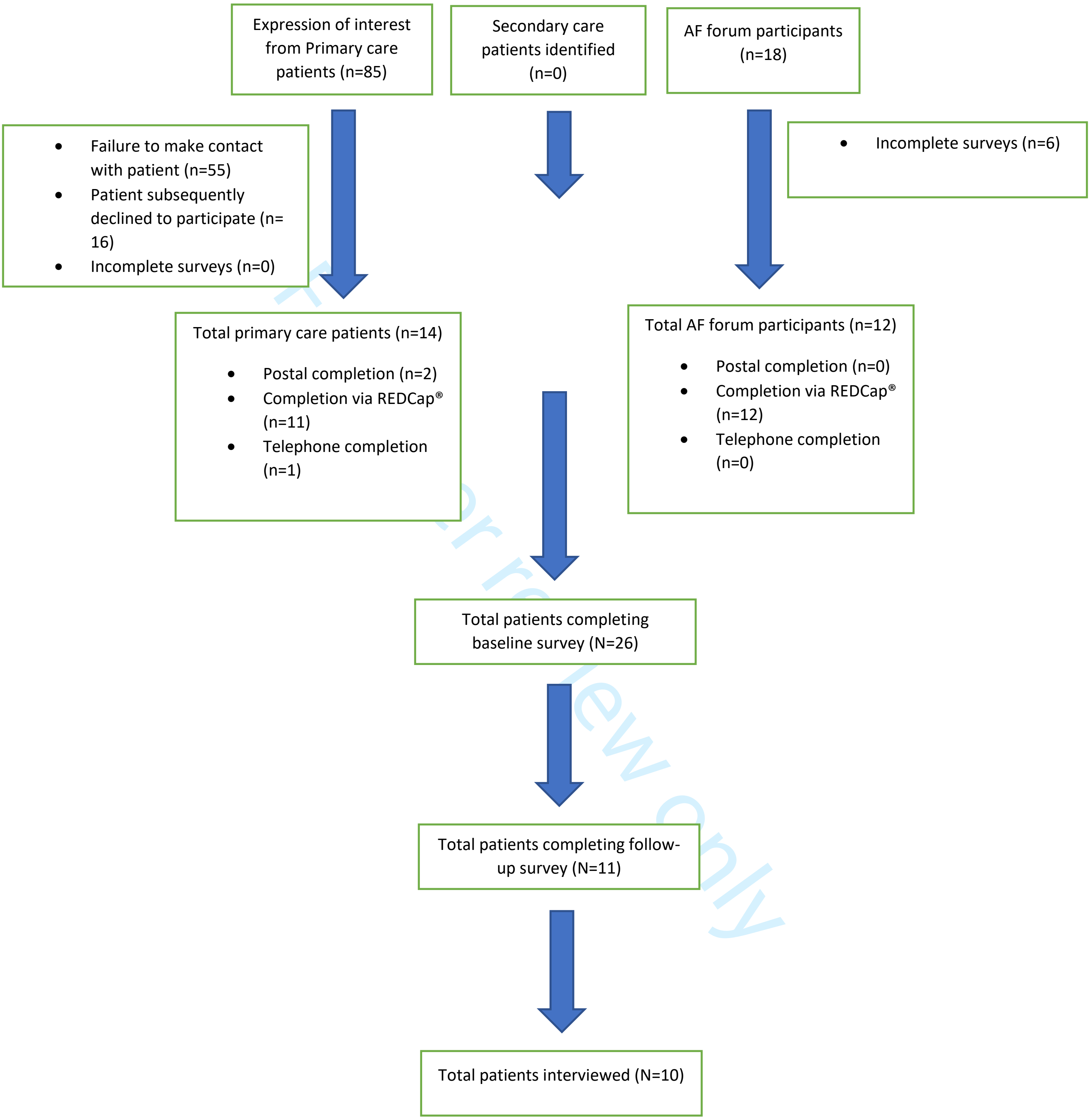

STROBE Statement—Checklist of items that should be included in reports of ***cross-sectional studies***

	Item No	Recommendation	Page No
**Title and abstract**	1	(*a*) Indicate the study's design with a commonly used term in the title or the abstract	1
		(*b*) Provide in the abstract an informative and balanced summary of what was done and what was found	2-3
**Introduction**			
Background/rationale	2	Explain the scientific background and rationale for the investigation being reported	5
Objectives	3	State specific objectives, including any prespecified hypotheses	5
**Methods**			
Study design	4	Present key elements of study design early in the paper	8
Setting	5	Describe the setting, locations, and relevant dates, including periods of recruitment, exposure, follow-up, and data collection	7
Participants	6	(*a*) Give the eligibility criteria, and the sources and methods of selection of participants	6
Variables	7	Clearly define all outcomes, exposures, predictors, potential confounders, and effect modifiers. Give diagnostic criteria, if applicable	8
Data sources/ measurement	8*	For each variable of interest, give sources of data and details of methods of assessment (measurement). Describe comparability of assessment methods if there is more than one group	*8*
Bias	9	Describe any efforts to address potential sources of bias	7
Study size	10	Explain how the study size was arrived at	8
Quantitative variables	11	Explain how quantitative variables were handled in the analyses. If applicable, describe which groupings were chosen and why	8
Statistical methods	12	(*a*) Describe all statistical methods, including those used to control for confounding	8
		(*b*) Describe any methods used to examine subgroups and interactions	N/A
		(*c*) Explain how missing data were addressed	8
		(*d*) If applicable, describe analytical methods taking account of sampling strategy	N/A
		(*e*) Describe any sensitivity analyses	N/A
**Results**			
Participants	13*	(a) Report numbers of individuals at each stage of study—eg numbers potentially eligible, examined for eligibility, confirmed eligible, included in the study, completing follow-up, and analysed	10-11
		(b) Give reasons for non-participation at each stage	10
		(c) Consider use of a flow diagram	20
Descriptive data	14*	(a) Give characteristics of study participants (eg demographic, clinical, social) and information on exposures and potential confounders	22-23
		(b) Indicate number of participants with missing data for each variable of interest	22-23
Outcome data	15*	Report numbers of outcome events or summary measures	22-23

For peer review only

Main results	16	(*a*) Give unadjusted estimates and, if applicable, confounder-adjusted estimates and their precision (eg, 95% confidence interval). Make clear which confounders were adjusted for and why they were included	N/A
		(*b*) Report category boundaries when continuous variables were categorized	24-25
		(*c*) If relevant, consider translating estimates of relative risk into absolute risk for a meaningful time period	N/A
Other analyses	17	Report other analyses done—eg analyses of subgroups and interactions, and sensitivity analyses	N/A
**Discussion**			
Key results	18	Summarise key results with reference to study objectives	12
Limitations	19	Discuss limitations of the study, taking into account sources of potential bias or imprecision. Discuss both direction and magnitude of any potential bias	12-13
Interpretation	20	Give a cautious overall interpretation of results considering objectives, limitations, multiplicity of analyses, results from similar studies, and other relevant evidence	12-13
Generalisability	21	Discuss the generalisability (external validity) of the study results	12-13
**Other information**			
Funding	22	Give the source of funding and the role of the funders for the present study and, if applicable, for the original study on which the present article is based	14

*Give information separately for exposed and unexposed groups.

Note: An Explanation and Elaboration article discusses each checklist item and gives methodological background and published examples of transparent reporting. The STROBE checklist is best used in conjunction with this article (freely available on the Web sites of PLoS Medicine at http://www.plosmedicine.org/, Annals of Internal Medicine at http://www.annals.org/, and Epidemiology at http://www.epidem.com/). Information on the STROBE Initiative is available at www.strobe-statement.org.

For peer review only

BMJ Open

Can we collect health-related quality of life information from anticoagulated atrial fibrillation participants who have recently experienced a bleed? An observational feasibility study in primary, and secondary care in Wales and through a UK online forum

Journal:	*BMJ Open*
Manuscript ID	bmjopen-2023-075335.R2
Article Type:	Original research
Date Submitted by the Author:	12-Sep-2023
Complete List of Authors:	Hutchings, Hayley; Swansea University, Swansea University Medical School<br>Lanyon, Kirsty; Swansea University, Swansea University Medical School<br>Holland, Gail; Swansea University, Swansea University Medical School<br>Alikhan, Raza; University Hospital of Wales, Department of Haematology<br>Jenkins, Rhys; Swansea City GP Cluster<br>LAING, HAMISH; Swansea University, VBHC Academy, School of Management<br>Hughes, Arfon; Swansea University<br>Lobban, Trudie; Arrythmia Alliance<br>Pollock, Kevin; Bristol-Myers Squibb Pharmaceuticals Ltd<br>Tod, Daniel; Swansea University, Swansea University Medical School<br>Lister, Steven; Bristol-Myers Squibb Pharmaceuticals Ltd
**Primary Subject Heading**:	Cardiovascular medicine
Secondary Subject Heading:	Haematology (incl blood transfusion), Health services research, Patient-centred medicine, Research methods
Keywords:	Feasibility Studies, Bleeding disorders & coagulopathies < HAEMATOLOGY, Anticoagulation < HAEMATOLOGY, Quality of Life

SCHOLARONE™
Manuscripts

For peer review only - http://bmjopen.bmj.com/site/about/guidelines.xhtml

I, the Submitting Author has the right to grant and does grant on behalf of all authors of the Work (as defined in the below author licence), an exclusive licence and/or a non-exclusive licence for contributions from authors who are: i) UK Crown employees; ii) where BMJ has agreed a CC-BY licence shall apply, and/or iii) in accordance with the terms applicable for US Federal Government officers or employees acting as part of their official duties; on a worldwide, perpetual, irrevocable, royalty-free basis to BMJ Publishing Group Ltd ("BMJ") its licensees and where the relevant Journal is co-owned by BMJ to the co-owners of the Journal, to publish the Work in this journal and any other BMJ products and to exploit all rights, as set out in our licence.

The Submitting Author accepts and understands that any supply made under these terms is made by BMJ to the Submitting Author unless you are acting as an employee on behalf of your employer or a postgraduate student of an affiliated institution which is paying any applicable article publishing charge ("APC") for Open Access articles. Where the Submitting Author wishes to make the Work available on an Open Access basis (and intends to pay the relevant APC), the terms of reuse of such Open Access shall be governed by a Creative Commons licence – details of these licences and which Creative Commons licence will apply to this Work are set out in our licence referred to above.

Other than as permitted in any relevant BMJ Author's Self Archiving Policies, I confirm this Work has not been accepted for publication elsewhere, is not being considered for publication elsewhere and does not duplicate material already published. I confirm all authors consent to publication of this Work and authorise the granting of this licence.

For peer review only

Can we collect health-related quality of life information from anticoagulated atrial fibrillation participants who have recently experienced a bleed? An observational feasibility study in primary, and secondary care in Wales and through a UK online forum

Hayley A Hutchings[1*], Kirsty Lanyon[1], Gail Holland[1], Raza Alikhan[2], Rhys Jenkins[3], Hamish Laing[4], Arfon Hughes[5], Trudie Lobban[6], Kevin G Pollock[7], Daniel Tod[1], Steven Lister[7]

1 Swansea University Medical School, Swansea University, Singleton Park, Swansea SA2 8PP, UK

*Corresponding author- Hayley A Hutchings

Email: h.a.hutchings@swansea.ac.uk

ORCID ID: https://orcid.org/0000-0003-4155-1741

2 Department of Haematology, University Hospital of Wales, Cardiff and Vale University Health Board, UK

3 Swansea City GP Cluster, Swansea, UK

4 VBHC Academy, School of Management, Swansea University

5 Public and Patient Involvement Representative

6 Arrhythmia Alliance, Public and Patient Representative, UK

7 Bristol Myers Squibb, Uxbridge Business Park, Sanderson Road, Uxbridge, Middlesex UB8 1DH, UK

Running head: health-related quality of life after a bleed

For peer review only - http://bmjopen.bmj.com/site/about/guidelines.xhtml

For peer review only

ABSTRACT

Objective

To evaluate the feasibility of recruiting participants diagnosed with atrial fibrillation (AF) taking oral anticoagulation therapies (OACs) and recently experiencing a bleed to collect health-related quality of life (HRQoL) information.

Design

Observational feasibility study. The study aimed to determine the feasibility of recruiting participants with minor and major bleeds, the most appropriate route for recruitment and the appropriateness of the Patient Reported Outcome Measures (PROMs) selected for collecting HRQoL information in AF patients, and the preferred format of the surveys.

Setting

Primary care, secondary care, and via an online patient forum.

Participants

The study population was adult patients (≥ 18) with Atrial Fibrillation (AF) taking oral anticoagulation therapies (OATs) who had experienced a recent major or minor bleed within the last four weeks.

Primary and Secondary outcome measures

Primary outcome:

Patient reported outcome measures (PROMs): EuroQol 5 dimensions-5 levels (EQ-5D-5L); Perception of anticoagulant treatment questionnaire, part 2 only (PACT-Q, part 2); Atrial fibrillation effect on quality of life (AFEQT)

Secondary outcomes:

Location of bleed; bleed severity; current treatment; patient perceptions of HRQoL in relation to bleeding events.

Results

We received initial expressions of interest from 103 participants. We subsequently recruited 32 participants to the study- 14 from primary care and 18 through the AF forum. No participants were recruited through secondary care. Despite 32 participants consenting, only 26 initial surveys were completed. We received follow-up surveys from 11 participants (8 primary care and 3 AF forum). COVID-19 had a major impact on the study.

Conclusions

Primary care was the most successful route for recruitment. Most participants recruited to the study experienced a minor bleed. Further ways to recruit in secondary care should be explored, especially to capture more serious bleeds.

Registration

For peer review only

The study was adopted onto the NIHR Portfolio (I.D. #47771) and registered with www.ClinicalTrials.gov (#NCT04921176) in February 2021.

Strengths and limitations of this study

- Multiple routes were used to identify potential participants with AF who had experienced a recent bleed- primary care, secondary care and through an online AF forum.
- Participants were able to complete the survey online (via REDCap®), on a paper-based form or over the telephone.
- Three validated patient reported outcome measures (PROMs) were used to gather different aspects of the impact of bleeding on AF patients.
- COVID- 19 impacted on the recruitment of participants and alternative mechanisms for recruitment need to be considered.

Key words: anticoagulation; bleeding disorders & coagulopathies; feasibility studies; health-related quality of life

Word count (excluding title page, abstract, references, figures, tables or acknowledgments)- 4072

For peer review only - http://bmjopen.bmj.com/site/about/guidelines.xhtml

INTRODUCTION

Atrial Fibrillation (AF) is the most common cardiac arrythmia (irregular heart rhythm disorder). Estimated overall prevalence has been reported to be between 0.4-1%[1, 2]. UK estimates have suggested that AF prevalence is increasing and could be as high as 3.29%[3]. Many clinicians however debate the true global prevalence, believing that it is likely to be underestimated as many individuals are asymptomatic and are likely to go undiagnosed[4]. The rate increases with age over 60 years[5] and it is estimated to be much higher in individuals over 80 and in Western societies, with suggested rates as high as 14%[6]. There are known to be high numbers of individuals with undiagnosed AF[7-9].

AF is a chronic condition associated with significant risks of thromboembolism, stroke, and mortality[10, 11]. Oral anticoagulation therapies (OATs)[1] are normally prescribed to reduce the risks of thromboembolism and AF-related stroke, but are associated with the side effect of bleeding, which in some cases can be serious[10, 12, 13]. These bleeding complications can include intracranial haemorrhage[14], gastrointestinal bleeding, and haematuria[15]. Bleeding as a side effect of OATs[16] remains a major challenge for clinicians that can often result in non-adherence and/or resistance to therapy[17]. While hospitalized AF-related stroke rates have declined and are significantly associated with increased anticoagulant uptake[18], the increased use of OATs has led to an increase in the number of bleeding events over time[10].

Patients are treated by healthcare providers with the primary goal of improving their health and well-being. Historically this improvement in health has been judged by improvement in biochemical, histological, radiological or clinical assessments. It is hypothesised that this approach does not always correlate with improvement from the patient perspective[19]. From a patient perspective, improving health is reflected in the documentation of their symptoms and experience of healthcare provision, which are more appropriately collected directly from the patient[20]. With a move towards shared-decision making and patient-centred care there is growing recognition within the healthcare community of the importance of the patient perspective and the need to consider patient-reported outcome measures (PROMs) as a key component of a holistic approach to patient care.

PROMs were initially developed for research use, and many regulatory authorities such as the European Medicines Agency (EMA) and the United States (US) Food and Drug Administration (FDA) advocate their use[21-23]. The U.S. Food and Drug Administration (FDA) defines a PROM as "any report of the status of a patient's health condition that comes directly from the patient, without

For peer review only - http://bmjopen.bmj.com/site/about/guidelines.xhtml

interpretation of the patient's response by a clinician or anyone else"[22]. The European Medicines Agency state that "Any outcome evaluated directly by the patient himself and based on the patient's perception of a disease and its treatment(s) is called a patient-reported outcome (PRO)"[24]. PROMs include quality of life (QoL) measures, disease severity scales, and patient experience measures.

The collection of PROMs aligns well with the increased drive within healthcare organisations for Value-Based healthcare, where organisations aim to achieve the best possible outcomes for patients with the available resources[25, 26]. As more clinicians recognise the benefit of collecting PROMs in addition to measuring clinical outcomes, PROMs have seen an increased use in routine clinical practice[27].

AF is thought to have a detrimental effect on health-related quality of life (HRQoL) in patients [28-33]. This research has focused on exploring HRQoL following various treatments or procedures[34-36], the development of AF specific HRQoL measures[37-40], or the use of existing HRQoL measures in AF populations[41, 42]. There is some evidence that bleeding events may have an adverse effect on patient HRQoL [43, 44] but there has been limited exploration of HRQoL following a bleed in AF patients[42, 45]. The aim of this study was to determine the feasibility of collecting HRQoL information from anticoagulated AF participants who had recently experienced a bleed. If this proves feasible, we plan to embark on a fully-powered study to evaluate the different dimensions of HRQoL of AF patients following a bleed.

METHODS

Selection of PROM to assess HRQoL

Prior to identifying sites and recruitment of participants, we undertook a process of PROM selection in order to select suitable tools that would allow us to assess HRQoL in AF participants who had experienced a recent bleed (reported elsewhere). This included a review of the literature to identify generic and disease specific PROMs that had been used in AF participants. Following the compilation of a list of possible PROMs, we undertook a rigorous evaluation of the identified PROMs that included an assessment of the overall validity of the PROMs, their content validity in terms of appropriateness of questions relating to bleeding events with AF, and how widely the PROM had previously been used in AF. This was informed by existing literature[46, 47] and included a clear justification as to why the PROM was used[48]. In selecting the PROMs, we were also mindful to ensure the burden to participants was kept to a minimum, so also explored the number of questions within relevant PROMs.

For peer review only - http://bmjopen.bmj.com/site/about/guidelines.xhtml

We worked with the two Public and Patient Involvement representatives when assessing the PROMs for help in considering their usefulness to the study.

Following the PROM scoping process, we agreed on three PROMs for use in the study:

- Euroquol 5 Dimensions-5 Levels (EQ-5D-5L)[49],

The EQ-5D is made up of the EQ-5D UK crosswalk scores which ranges between -0.594 to 1.000. A higher index score indicates a better QoL. The EQ-5D VAS (visual analogue scale) allows users to input a score of how good or bad their health is between 0 to 100, with a higher VAS score indicating a better QoL.

- Perception of Anticoagulant Treatment Questionnaire, Part 2 only (PACT-Q, Part 2)[50, 51]; and

The PACT-Q Part 2 scores range from 0 to 100, for the convenience and satisfaction domains, with a higher score indicating higher convenience and higher satisfaction.

- Atrial fibrillation Effect on QualiTy-of-life (AFEQT)[41].

The disease specific AFEQT global and specific domain scores range from 0 to 100. A score of 0 corresponds to complete disability, while a score of 100 corresponds to no disability.

Participant Recruitment

We recruited adult participants (aged ≥18 years) who had a diagnosis of AF and were actively prescribed OATs for their AF. We identified participants who had experienced a recent bleed, up to a maximum of 30 days prior to enrolment. No upper age limit was imposed, but participants had to fit all other inclusion and exclusion criteria.

Inclusion criteria

- Adult participants (≥18 years)
- Participants who had a fluent understanding of English and were able to comprehend all study information and literature to provide fully informed consent
- Atrial fibrillation (AF) as the primary diagnosis
- Had a major or minor bleed up to a maximum of 30 days prior to date of enrolment
- Prescribed oral anticoagulation for AF

Exclusion Criteria

- Pregnant women
- Participants with active cancer
- Participants unable to consent for themselves
- Participants on concomitant antiplatelet therapy

For peer review only - http://bmjopen.bmj.com/site/about/guidelines.xhtml

As the study was designed to test feasibility for a fully powered definitive study, we aimed to recruit between 50 and 80 participants in the first instance based on recommended sample size guidance[52]. Figure 1 outlines the stages of the study.

Participating sites

For this feasibility study, we selected participants from the Swansea, UK area. We utilised three recruitment pathways for the study to test the feasibility of identifying and recruiting participants with minor and major bleeds:

- Primary care – Participants attending anticoagulation clinics of a large city General Practice cluster made up of 8 practices and serving almost 51,000 patients in Swansea, UK. Participants were identified by practice nurse/pharmacist as having a recent bleed.
- Secondary care – Participants admitted to the emergency department of a large UK University teaching hospital in Swansea, UK with a bleed were identified by research nurses/secondary care-based general practitioner.
- Direct participant – The study was publicised on the Arrhythmia Alliance website and newsletters (Arrhythmia Alliance UK (heartrhythmalliance.org) and individuals who formed part of this online forum and database were invited to participate.

When participants were identified through primary and secondary care, they were provided with a study information sheet for their consideration. If the patient was willing to participate, they were asked to return the expression of interest form to the study researcher in the supplied freepost envelope. The study researcher then contacted participants to provide them with a 'study pack'. As we aimed to determine the best approach for collecting the HRQoL information, we offered the participants the option of an electronic link to a REDCap® survey, a paper survey sent to their home with a freepost envelope, or the option to complete the survey by telephone. Individuals from the AF forum were initially provided with a weblink to the REDCap® survey through the website. Latterly, they were given the option to contact the researcher and be supplied with a paper survey.

For peer review only - http://bmjopen.bmj.com/site/about/guidelines.xhtml

Participants Survey Pack

We collected basic demographics from participants, details of their current OAT, existing co-morbidities, and details of any bleed(s) they had experienced (i.e. date, severity and bleed location). We asked all participants to complete three PROMs for the study survey at baseline: the Euroquol 5 Dimensions-5 Levels (EQ-5D-5L)[49], Perception of Anticoagulant Treatment Questionnaire (PACT-Q) Part 2[50, 51], and the Atrial fibrillation Effect on QualiTy-of-life (AFEQT)[41].

We estimated that completion of all study questionnaires would take no longer than 30 minutes. Participants who agreed to participate were asked to complete the survey at two time points - once following consent, and then again at 90±14 days post-enrolment. Participants who completed the survey digitally via the REDCap® system received a second online link to complete the survey; those who completed paper forms were sent a follow up via post (along with a stamped addressed envelope for ease of return); and those who completed over the phone were contacted via telephone to complete the follow-up. Participants were contacted 3 weeks prior to the 90-day follow-up date. If no reply was received within 2 weeks of first correspondence, a reminder letter (with a second copy of all questionnaires) was sent, or a follow-up email or telephone call was made.

Data analysis

Quantitative data were checked, cleaned, and collated in REDCap® prior to being transferred to Stata Version 17.0.for analyses. As this was a feasibility study and not powered to detect differences between groups/OATs, no formal statistical comparisons were made. Data are presented descriptively as number, percentages, or means (standard deviation). HRQoL scores were calculated according to developers' guidance. Descriptive HRQoL scores are reported for AF participants following a minor or major bleed[53, 54] while anticoagulated and by recruitment route. Categorisation of the bleed into minor or major was undertaken by a clinical professional (RA).

Ethics

We complied with Good Clinical Practice (GCP) guidelines and the General Data Protection Regulation (GDPR) in the conduct of the study and the collection of participant information. The study was given ethical approval from the London - Riverside Research Ethics Committee Research Ethics Committee (IRAS ID 279646), and Swansea Bay University Health Board R&D. The study research governance sponsor was Swansea University.

For peer review only - http://bmjopen.bmj.com/site/about/guidelines.xhtml

Participants were required to provide consent before enrolment into the study. Prior to consent we provided participants with an information sheet which ensured that they were clearly and fully informed about the purpose of the study, potential risks, use of their data, and their rights and responsibilities when participating in the study. They were also told that they had the right to withdraw at any point in the study. Participants who completed the survey via the REDCap® system consented to the study electronically. Participants completing paper forms were required to complete a paper consent and we obtained verbal consent for those completing over the telephone. We asked participants for informed consent for their anonymised data and interview quotes (where relevant) to be used in any future publications.

We were aware that participants may have experienced low mood or anxiety associated with their AF. If a participant reported, or showed signs of low mood, distress or anxiety, we encouraged them to discuss this with their primary or secondary care researcher and the participant was signposted to local relevant services or advised to contact their GP.

We stored all data securely within the password protected REDCap® secure web application while the study was conducted, and subsequently anonymised for analysis purposes.

COVID implications/changes

This study was originally conceived and designed before the COVID-19 pandemic. Shortly after receiving ethical approval and prior to the start of recruitment, the country went into its first lockdown to prevent onward transmission of the virus. Initially, the study was paused as we knew that participant recruitment through primary and secondary care would be impacted. As time progressed, however, we decided to adapt the study to take account of the situation and the change in operating practices across sites. The initial protocol included processes for face-to-face recruitment in primary care (through routine anticoagulation clinics) and in secondary care (through identification of AF bleeding cases through medical records and approach of patients on the wards).

To minimise any potential COVID-19 risk to both research staff and patients, it was agreed therefore that the study design should operate in line with current guidelines set out by the UK and Welsh Government for COVID-19 management. Up-to-date Government guidance was therefore followed throughout the study. This had a direct consequence on some aspects of study conduct. Study visits were therefore changed to ensure that they could be conducted remotely, until such time where face-to-face visits were permitted. Key members of the study team monitored relevant guidance for GP

For peer review only - http://bmjopen.bmj.com/site/about/guidelines.xhtml

For peer review only

surgeries and hospitals closely and necessary adjustments to study conduct were made accordingly, and consistently with any new information and/or restrictions as it was released. Where study tasks were conducted remotely, Health Research Authority (HRA) advice released for conducting research during the COVID-19 pandemic was followed, where applicable. Any study visits conducted in person took place with all required precautions in place to help prevent the spread of the virus.

Patient and Public Involvement (PPI)

We recruited two PPI representatives prior to initiating the study. They had an equal voice on our study steering committees and attended all meetings. They provided input on study design, selection of appropriate data collection methods/tools, development of all patient facing documents and the ethics application. Our PPI's were selected based on appropriate relatedness to the condition, i.e. living with AF and prescribed anticoagulants and/or those involved with AF support groups. Our PPI's reviewed this manuscript prior to submission and are co-authors on the publication.

RESULTS

We received initial expressions of interest from 85 participants in primary care. These expressions of interest were however not translated into participation in most cases. 31 questionnaires were sent out following successful initial contact with prepaid addressed envelopes for return but no further contact with the participant could be made. In addition, 18 participants started the survey on the AF forum. A total of 32 participants consented to participate in the study - 14 from primary care and 18 through the AF forum. No participants were recruited through secondary care.

COVID-19 had a major impact on the study, particularly in secondary care. We had difficulty recruiting in secondary care for three reasons. Firstly, many research nurses were transferred back to help manage frontline NHS activities; secondly, those who were still active had difficulty gaining access to participants and wards during lockdown; and thirdly, Welsh Government mandated that research nurse activity should be prioritised to COVID-19 vaccine studies. As a result, we focused on the two other recruitment routes, primary care and the AF forum.

Despite 32 participants consenting to participate, only 26 initial surveys were completed (14 from primary care and 12 from the AF forum), with 6 from the AF forum being abandoned prior to completion of any questions on REDCap®. We contacted primary care contacts at least twice if we had received an expression of interest from them.

For peer review only - http://bmjopen.bmj.com/site/about/guidelines.xhtml

We received follow-up surveys from 11 participants (8 primary care and 3 AF forum). Figure 2 illustrates the numbers of participants expressing an interest and subsequently consenting to participate in the study.

Table 1 illustrates the responses to the general questions from the 26 participants in the primary care and AF forum groups. We only had demographic information for the primary care group, where clinical data were cross referenced to patient records. The primary care patients were all white and mostly female (79%). The HASBLED score (all less than 3) indicated that participants were at low risk of bleeding.

In both the primary care and AF forum groups, participants reported many pre-existing conditions. The most common were hypertension (reported by 43% of participants) and other (64%). There were a range of times since the bleed, including beyond 30 days, despite recruitment aiming to focus on bleeds of 30 days or less. Minor bleeding was more common than major bleeding in both groups, with 6/14 (42.9%) of participants in the primary care group and 5/12 (41.7%) in the AF forum group indicating that they had fairly frequent minor bleeds (1-2 per week or 1-2 per month). This was in contrast to the frequency of major bleeds, where 10/14 (71.4%) of participants from primary care and 7/12 (58.3%) from the AF forum indicated that they almost never had major bleeds. Clinical classification of the most recent bleed (where documented) indicated that there were 18 minor, 2 major and 1 unknown.

Table 2 illustrates the HRQoL scores from the three questionnaires. The mean EQ-5D UK crosswalk and EQ-5D VAS scores in the primary care group were lower than that in the AF forum group at baseline (0.470 vs 0.809 crosswalk; 58.9 vs 70.6 VAS). The follow up scores for both the cross walk and VAS scores in the primary care group were higher, but still less than the AF forum group (0.694 vs 0.893 crosswalk; 60.1 vs 83.0 VAS).

The AFEQT overall and subscale scores were largely in the positive range indicating limited disability due to AF. At baseline, the global AFEQT score, symptom, daily activities, and treatment concern domain scores in the primary care and AF forum group were similar for the global, symptom and daily activities scores (64.8 vs 65.7 global; 76.4 vs 78.0 symptom; 50.0 vs 58.7 daily activities). The participants in the primary care group had less treatment concern at baseline that the AF forum group (76.9 vs 66.9; treatment concern). The global and symptom scores showed little change between the

For peer review only - http://bmjopen.bmj.com/site/about/guidelines.xhtml

baseline and follow-up survey in the primary care group (64.8 vs 63.3 global; 76.4 vs 74.0). The primary care group score did however improve for daily activities (50.0 vs 59.8; daily activities) and deteriorate for treatment concern (76.9 vs 59.1). In the AF forum group, there was an improvement in the global score (65.7 vs 77.2 global) and little change in the symptom score (78.0 vs 81.9 symptom). There was also an improvement in the daily activities (58.7 vs 76.4 daily activities) and treatment concern (66.9 vs 75.0) score in the AF group between baseline and follow-up. Both groups had a deterioration in response to the two AFEQT satisfaction questions (Q19 and 20, see Table 2). These questions relate to how well they think their current treatment controls their AF and the extent to which their treatment has relieved their symptoms.

The PACT-Q2 convenience scores were comparable between the primary care and AF forum groups at baseline (87.0 vs 86.5 convenience). There was little change in these scores at follow-up in the primary care (87.0 vs 86.8) or the AF forum group (86.5 vs 86.5). The initial PACT-Q2 satisfaction scores were again comparable between the primary care and AF forum group (65.4 vs 67.5 satisfaction). The primary care group however showed a deterioration in the satisfaction score at follow-up (65.4 vs 61.6), whereas the AF forum group showed an improvement in scores (67.5 vs 76.2) at follow-up.

Due to the small numbers participating in the surveys, it is difficult to infer any meaningful conclusions from these results and as such they should be interpreted with caution.

DISCUSSION

After receiving the necessary ethics and R&D approvals for the study, the UK went into lockdown, and we were forced to initially pause the study. When we were in a position to restart, we had to modify the study to take account of changing healthcare practices. This included remote engagement and varying formats for completion of the questionnaires. We used various approaches to increase recruitment, including the option to complete the survey on paper, through an online survey link or over the phone. Despite receiving many expressions of interest and making multiple phone calls, we were unable to make contact with most patients who had expressed an interest in participating. The study was open for a year longer than planned (2 years in total) after which the research management group made the decision to close.

Recruitment in secondary care was mostly impacted and we were unable to recruit any patients through this route. This was largely due to nurses being reassigned to front-line roles, limited access

For peer review only

For peer review only - http://bmjopen.bmj.com/site/about/guidelines.xhtml

to wards, and a focus on recruitment to Covid-19 studies. We were still able to recruit through primary care and the AF online forum. There were however a number of incomplete questionnaires submitted through the online forum. This may indicate that patients find online forms difficult to complete[55] or that they were unsure of the relevance of the questions or found the survey too long. Separate qualitative interviews with patients will explore these concepts further.

Primary care therefore appeared to be the best way to recruit participants. This may have been because they were introduced to the study by the primary care pharmacist whom they knew, and they were therefore not contacted 'cold'. We received initial expressions of interest in primary care from 90 participants. This did not however translate into 90 completed questionnaires. This may be because of the time lapse between discussing the study and subsequently being contacted by the researcher or misunderstanding regarding the rationale for the study. Had the participants received the questionnaire on initial contact, this may have increased the completion rate.

Most of the patients that completed the survey had minor bleeds. This may be due to the fact that most patients were recruited through primary care. Had we recruited through secondary care, we may have seen more major bleeds.

Selecting the PROMs for the study was challenging. The focus of the study was to test the feasibility of collecting data regarding HRQoL in AF participants on OATs who experienced a bleed. Details of the selection process for the PROMs will be reported separately, but the final decision was based on 1) previous use of the PROMs with the AF population; 2) assessment of the validity of the PROMs; and 3) relevance of the questions to a bleeding event. There were few PROMs that had questions that specifically focused on the bleed. This may have also contributed to the fact that the response rate was lower than expected.

It was difficult to interpret the HRQoL scores as the study was not powered to detect any statistically significant differences. In addition, there was a great deal of variability in the scores across the different scales even within the same groups. Due to the limited number of participants recruited and the variability in the scores obtained, contextualising these scores with existing literature is therefore difficult. Further studies should explore whether the type of OAT has any impact on HRQoL, particularly as some OATs are less likely to cause bleeding. We collected information regarding existing co-morbidities and a future study should also explore whether any of these conditions are likely to affect the risk of bleeding.

For peer review only - http://bmjopen.bmj.com/site/about/guidelines.xhtml

Further work needs to be undertaken to explore alternative ways of recruiting through secondary care. In our study we utilised research nurses with the aim of accessing ward admission records to identify potential patients. This mechanism for recruitment was particularly impacted by the pandemic; access to the wards was restricted, many research nurses were moved back to frontline activities, and covid-19 research studies were prioritised. Recruitment through secondary care may be improved by working directly with clinicians who manage AF patients on OATs. It is likely that this would also increase the number of participants recruited with major bleeds and help to determine the most appropriate time to approach participants, especially as they may be too ill during their inpatient episode.

The way we recruited through primary care could also be streamlined, with participants being invited to participate following their initial contact with the lead clinician managing the bleeding clinics rather than being contacted by the researcher following completion of an expression of interest. The relevance of the questionnaires may have impacted on the completion of the survey through the online forum, so use of a more specific PROM that focused on bleeding issues may facilitate this.

Despite the challenges of undertaking the study in the pandemic, we were still able to recruit 32 patients. A further fully- powered study is now needed to explore whether HRQoL is impacted by bleeding episodes in AF patients on OATs, and whether the extent of the bleed has an effect on HRQoL.

AUTHOR CONTRIBUTIONS

HAH and SL conceived the study. HAH designed the initial study proposal, led on interpretation of the findings and drafted the manuscript. KL was responsible for site set-up, ethical approvals, participant recruitment and data collection; participated in interpretation of findings and provided input on the manuscript. GH provided overall study governance support, specifically in relation to ethical aspects, provided input into final protocol, participated in interpretation of findings and provided input on the manuscript. RA provided input into final protocol, was responsible for clinical interpretation of bleeds, participated in interpretation of findings and provided input on the manuscript. RJ led primary care recruitment, provided input into final protocol, participated in interpretation of findings and provided input on the manuscript. TL led AF forum recruitment, provided input into final protocol, provided PPI input on survey design, participated in interpretation of findings and was provided input on the manuscript. AH provided input into final protocol, provided PPI input on survey design, participated in interpretation of findings and provided input on the manuscript. DT was responsible for writing the

For peer review only - http://bmjopen.bmj.com/site/about/guidelines.xhtml

For peer review only

analysis plan, final analysis and interpretation of the findings and provided input on the manuscript. HL, KP provided input into final protocol, participated in interpretation of findings and provided input on the manuscript. All authors reviewed and approved the final manuscript.

ACKNOWLEDGEMENTS

We would like to thank all participants for completing the survey, the general practice nurses from Swansea Bay City Cluster who helped with recruitment, and the Arrhythmia Alliance for hosting the online survey.

FUNDING AND GOVERNANCE

The Chief Investigator of the study was Professor Hayley Hutchings, Swansea Trials Unit, Swansea University (SU). Under the UK Policy Framework for Health and Social Care Research SU met the requirements of research governance sponsor. BMS/Pfizer funded the research (grant number: CV185-770) and under the UK policy framework were part of a partnership that accepted overall responsibility for proportionate and effective arrangements to be in place to set up, run and report the research project. They were represented on the Trial Steering Committee throughout the project.

CONFLICT OF INTEREST

Hayley A Hutchings leads this independent research which was funded by Pfizer and Bristol Myers Squibb.

Hamish Laing is an employee of Swansea University who has received a grant for a Value-Based Healthcare programme of work from Pfizer Ltd., which he leads and support to attend a meeting.

Steven Lister was an employee of BMS at the time of the study and holds stock in BMS and Pfizer.

Kevin Pollock is an employee of BMS and holds stock in BMS.

Gail Holland, Kirsty Lanyon, Arfon Hughes, Rhys Jenkins, Trudie Lobban, declare that they have no conflict of interest.

DATA SHARING AGREEMENT

The anonymised dataset are available on the Zenodo open access data repository.

For peer review only

For peer review only - http://bmjopen.bmj.com/site/about/guidelines.xhtml

REFERENCES

1. Liu GJ, Wang YF, Chen PY, Chang W, Tu ML, Chang LY, et al. The efficacy and safety of novel oral anticoagulants for the preventive treatment in atrial fibrillation patients: a systematic review and meta-analysis. Drug Deliv. 2014;21(6):436-52. doi: 10.3109/10717544.2013.873500. PubMed PMID: 24400656.
2. Zulkifly H, Lip GYH, Lane DA. Epidemiology of atrial fibrillation. Int J Clin Pract. 2018;72(3):e13070. Epub 2018/03/02. doi: 10.1111/ijcp.13070. PubMed PMID: 29493854.
3. Adderley NJ, Ryan R, Nirantharakumar K, Marshall T. Prevalence and treatment of atrial fibrillation in UK general practice from 2000 to 2016. Heart. 2019;105(1):27-33. Epub 2018/07/12. doi: 10.1136/heartjnl-2018-312977. PubMed PMID: 29991504.
4. Patel NJ, Atti V, Mitrani RD, Viles-Gonzalez JF, Goldberger JJ. Global rising trends of atrial fibrillation: a major public health concern. Heart. 2018;104(24):1989-90. Epub 2018/06/17. doi: 10.1136/heartjnl-2018-313350. PubMed PMID: 29907645.
5. Darby-Stewart A, Dachs R, Graber MA. Rivaroxaban vs. warfarin for stroke prevention in patients with nonvalvular atrial fibrillation. Am Fam Physician. 2012;85(6):577-86. PubMed PMID: 22534269.
6. Kodani E, Atarashi H. Prevalence of atrial fibrillation in Asia and the world. Journal of Arrhythmia. 2012;28(6):330-7.
7. Turakhia MP, Shafrin J, Bognar K, Trocio J, Abdulsattar Y, Wiederkehr D, et al. Estimated prevalence of undiagnosed atrial fibrillation in the United States. PLoS One. 2018;13(4):e0195088. Epub 2018/04/13. doi: 10.1371/journal.pone.0195088. PubMed PMID: 29649277; PubMed Central PMCID: PMCPMC5896911.
8. Clua-Espuny JL, Lechuga-Duran I, Bosch-Princep R, Roso-Llorach A, Panisello-Tafalla A, Lucas-Noll J, et al. Prevalence of undiagnosed atrial fibrillation and of that not being treated with anticoagulant drugs: the AFABE study. Rev Esp Cardiol (Engl Ed). 2013;66(7):545-52. Epub 2014/04/30. doi: 10.1016/j.rec.2013.03.003. PubMed PMID: 24776203.
9. Suissa L, Lachaud S, Mahagne MH. Optimal timing and duration of continuous electrocardiographic monitoring for detecting atrial fibrillation in stroke patients. J Stroke Cerebrovasc Dis. 2013;22(7):991-5. Epub 2012/02/22. doi: 10.1016/j.jstrokecerebrovasdis.2012.01.015. PubMed PMID: 22349706.
10. Hansen PW, Sehested TSG, Fosbol EL, Torp-Pedersen C, Kober L, Andersson C, et al. Trends in warfarin use and its associations with thromboembolic and bleeding rates in a population with atrial fibrillation between 1996 and 2011. PLoS One. 2018;13(3):e0194295. Epub 20180316. doi: 10.1371/journal.pone.0194295. PubMed PMID: 29547673; PubMed Central PMCID: PMCPMC5856343.
11. Kannel WB, Wolf PA, Benjamin EJ, Levy D. Prevalence, incidence, prognosis, and predisposing conditions for atrial fibrillation: population-based estimates. Am J Cardiol. 1998;82(8A):2N-9N. doi: 10.1016/s0002-9149(98)00583-9. PubMed PMID: 9809895.
12. Turagam MK, Parikh V, Afzal MR, Gopinathannair R, Lavu M, Kanmanthareddy A, et al. Replacing warfarin with a novel oral anticoagulant: Risk of recurrent bleeding and stroke in patients with warfarin ineligible or failure in patients with atrial fibrillation (The ROAR study). J Cardiovasc Electrophysiol. 2017;28(8):853-61. Epub 20170608. doi: 10.1111/jce.13254. PubMed PMID: 28497899.
13. Lamberts M, Staerk L, Olesen JB, Fosbol EL, Hansen ML, Harboe L, et al. Major Bleeding Complications and Persistence With Oral Anticoagulation in Non-Valvular Atrial Fibrillation: Contemporary Findings in Real-Life Danish Patients. J Am Heart Assoc. 2017;6(2). Epub 20170214. doi: 10.1161/JAHA.116.004517. PubMed PMID: 28196815; PubMed Central PMCID: PMCPMC5523754.
14. Hankey GJ. Intracranial hemorrhage and novel anticoagulants for atrial fibrillation: what have we learned? Curr Cardiol Rep. 2014;16(5):480. doi: 10.1007/s11886-014-0480-9. PubMed PMID: 24643903.

For peer review only - http://bmjopen.bmj.com/site/about/guidelines.xhtml

15. Wallis CJD, Juvet T, Lee Y, Matta R, Herschorn S, Kodama R, et al. Association Between Use of Antithrombotic Medication and Hematuria-Related Complications. JAMA. 2017;318(13):1260-71. doi: 10.1001/jama.2017.13890. PubMed PMID: 28973248; PubMed Central PMCID: PMCPMC5818855.
16. Khan F, Huang H, Datta YH. Direct oral anticoagulant use and the incidence of bleeding in the very elderly with atrial fibrillation. J Thromb Thrombolysis. 2016;42(4):573-8. doi: 10.1007/s11239-016-1410-z. PubMed PMID: 27520093; PubMed Central PMCID: PMCPMC5042846.
17. Dar T, Yarlagadda B, Vacek J, Dawn B, Lakkireddy D. Management of Stroke risk in atrial fibrillation patients with bleeding on Oral Anticoagulation Therapy-Role of Left Atrial Appendage Closure, Octreotide and more. J Atr Fibrillation. 2017;10(4):1729. doi: 10.4022/jafib.1729. PubMed PMID: 29487685; PubMed Central PMCID: PMCPMC5821636.
18. Cowan JC, Wu J, Hall M, Orlowski A, West RM, Gale CP. A 10 year study of hospitalized atrial fibrillation-related stroke in England and its association with uptake of oral anticoagulation. Eur Heart J. 2018;39(32):2975-83. Epub 2018/07/10. doi: 10.1093/eurheartj/ehy411. PubMed PMID: 29982405; PubMed Central PMCID: PMCPMC6110195.
19. Pakhomov SV, Jacobsen SJ, Chute CG, Roger VL. Agreement between patient-reported symptoms and their documentation in the medical record. Am J Manag Care. 2008;14(8):530-9. PubMed PMID: 18690769; PubMed Central PMCID: PMCPMC2581509.
20. Hutchings HA, Alrubaiy L. Patient-Reported Outcome Measures in Routine Clinical Care: The PROMise of a Better Future? Dig Dis Sci. 2017;62(8):1841-3. doi: 10.1007/s10620-017-4658-z. PubMed PMID: 28660488.
21. European Medicines Agency. Reflection paper on th regulatory guidance for the use of Health-Related Quality of Life (HRQL) measures in the evaluation of medicinal products. Available from: https://www.ema.europa.eu/en/documents/scientific-guideline/reflection-paper-regulatory-guidance-use-healthrelated-quality-life-hrql-measures-evaluation_en.pdf 2005 [5 November 2019].
22. U.S. Department of Health and Human Services, Food and Drug Administration, Center for Drug Evaluation and Research (CDER), Center for Biologics Evaluation and Research (CBER), Center for Devices and Radiological Health (CDRH). Guidance for Industry Patient-Reported Outcome Measures: Use in Medical Product Development to Support Labeling Claims. Available from: https://www.fda.gov/downloads/Drugs/GuidanceComplianceRegulatoryInformation/Guidances/UCM193282.pdf 2009 [5 November 2019].
23. Revicki DA, Regulatory I, Patient-Reported Outcomes Task Force for the International Society for Quality of Life R. FDA draft guidance and health-outcomes research. Lancet. 2007;369(9561):540-2. doi: 10.1016/S0140-6736(07)60250-5. PubMed PMID: 17307086.
24. Downing J, Namisango E, Harding R. Outcome measurement in paediatric palliative care: lessons from the past and future developments. Ann Palliat Med. 2018;7(Suppl 3):S151-S63. Epub 2018/09/06. doi: 10.21037/apm.2018.04.02. PubMed PMID: 30180720.
25. Porter ME, Larsson S, Lee TH. Standardizing Patient Outcomes Measurement. N Engl J Med. 2016;374(6):504-6. doi: 10.1056/NEJMp1511701. PubMed PMID: 26863351.
26. Porter ME, Lee TH. The Strategy That Will Fix Health Care. Harvard Bus Rev. 2013;91(12):24-. PubMed PMID: WOS:000327362300014.
27. Black N. Patient reported outcome measures could help transform healthcare. BMJ. 2013;346:f167. doi: 10.1136/bmj.f167. PubMed PMID: 23358487.
28. Peinado R, Arribas F, Ormaetxe JM, Badia X. Variation in quality of life with type of atrial fibrillation. Rev Esp Cardiol. 2010;63(12):1402-9. Epub 2010/12/15. doi: 10.1016/s1885-5857(10)70274-7. PubMed PMID: 21144400.
29. Zhang L, Gallagher R, Neubeck L. Health-related quality of life in atrial fibrillation patients over 65 years: A review. Eur J Prev Cardiol. 2015;22(8):987-1002. Epub 2014/06/14. doi: 10.1177/2047487314538855. PubMed PMID: 24924742.

For peer review only - http://bmjopen.bmj.com/site/about/guidelines.xhtml

30. Deaton C, Dunbar SB, Moloney M, Sears SF, Ujhelyi MR. Patient experiences with atrial fibrillation and treatment with implantable atrial defibrillation therapy. Heart Lung. 2003;32(5):291-9. Epub 2003/10/07. doi: 10.1016/s0147-9563(03)00074-8. PubMed PMID: 14528186.
31. Aliot E, Botto GL, Crijns HJ, Kirchhof P. Quality of life in patients with atrial fibrillation: how to assess it and how to improve it. Europace. 2014;16(6):787-96. Epub 2014/01/29. doi: 10.1093/europace/eut369. PubMed PMID: 24469433.
32. Ong L, Cribbie R, Harris L, Dorian P, Newman D, Mangat I, et al. Psychological correlates of quality of life in atrial fibrillation. Qual Life Res. 2006;15(8):1323-33. Epub 20060707. doi: 10.1007/s11136-006-0029-5. PubMed PMID: 16826433.
33. Thrall G, Lane D, Carroll D, Lip GY. Quality of life in patients with atrial fibrillation: a systematic review. Am J Med. 2006;119(5):448 e1-19. doi: 10.1016/j.amjmed.2005.10.057. PubMed PMID: 16651058.
34. Pokorney SD, Kim S, Thomas L, Fonarow GC, Kowey PR, Gersh BJ, et al. Cardioversion and subsequent quality of life and natural history of atrial fibrillation. Am Heart J. 2017;185:59-66. Epub 2017/03/08. doi: 10.1016/j.ahj.2016.10.018. PubMed PMID: 28267476.
35. Sun Y, Zhu J, Ma C, Liu S, Yang Y, Hu D. Stroke Risk Status, Anticoagulation Treatment, and Quality-of-Life in Chinese Patients with Atrial Fibrillation: China Registry of Atrial Fibrillation (CRAF). Cardiovasc Ther. 2019;2019:7372129. Epub 2019/11/28. doi: 10.1155/2019/7372129. PubMed PMID: 31772614; PubMed Central PMCID: PMCPMC6739760.
36. Bai Y, Bai R, Wu JH, Zhang T, Liu N, Shi XB, et al. Differences in Quality of Life Between Atrial Fibrillation Patients with Low Stroke Risk Treated With and Without Catheter Ablation. J Am Heart Assoc. 2015;4(9):e002130. Epub 2015/09/18. doi: 10.1161/JAHA.115.002130. PubMed PMID: 26376990; PubMed Central PMCID: PMCPMC4599501.
37. Coyne KS, Edvardsson N, Ryden A. Development and Validation of the AFImpact: An Atrial Fibrillation-Specific Measure of Patient-Reported Health-Related Quality of Life. Value Health. 2017;20(10):1355-61. Epub 2017/12/16. doi: 10.1016/j.jval.2017.06.005. PubMed PMID: 29241895.
38. Badia X, Arribas F, Ormaetxe JM, Peinado R, de Los Terreros MS. Development of a questionnaire to measure health-related quality of life (HRQoL) in patients with atrial fibrillation (AF-QoL). Health Qual Life Outcomes. 2007;5:37. Epub 2007/07/06. doi: 10.1186/1477-7525-5-37. PubMed PMID: 17610734; PubMed Central PMCID: PMCPMC1936414.
39. Arribas F, Ormaetxe JM, Peinado R, Perulero N, Ramirez P, Badia X. Validation of the AF-QoL, a disease-specific quality of life questionnaire for patients with atrial fibrillation. Europace. 2010;12(3):364-70. Epub 2010/01/09. doi: 10.1093/europace/eup421. PubMed PMID: 20056594.
40. Medin J, Arbuckle R, Abetz L, Halling K, Kulich K, Edvardsson N, et al. Development and validation of the AFSymp: an atrial fibrillation-specific measure of patient-reported symptoms. Patient. 2014;7(3):319-27. doi: 10.1007/s40271-014-0058-z. PubMed PMID: 24733480.
41. Spertus J, Dorian P, Bubien R, Lewis S, Godejohn D, Reynolds MR, et al. Development and validation of the Atrial Fibrillation Effect on QualiTy-of-Life (AFEQT) Questionnaire in patients with atrial fibrillation. Circ Arrhythm Electrophysiol. 2011;4(1):15-25. Epub 2010/12/17. doi: 10.1161/CIRCEP.110.958033. PubMed PMID: 21160035.
42. Wang K, Li H, Kwong WJ, Antman EM, Ruff CT, Giugliano RP, et al. Impact of Spontaneous Extracranial Bleeding Events on Health State Utility in Patients with Atrial Fibrillation: Results from the ENGAGE AF-TIMI 48 Trial. J Am Heart Assoc. 2017;6(8). Epub 2017/09/02. doi: 10.1161/JAHA.117.006703. PubMed PMID: 28862934; PubMed Central PMCID: PMCPMC5586476.
43. Res LCS, Lubberts B, Shah SH, DiGiovanni CW. Health-related quality of life after adverse bleeding events associated with antithrombotic drug therapy - A systematic review. Hellenic J Cardiol. 2019;60(1):3-10. doi: 10.1016/j.hjc.2018.06.012. PubMed PMID: 29908761.
44. Radaideh KM, Matalqah LM. Health-related quality of life among atrial fibrillation patients using warfarin therapy. Epidemiology Biostatistics and Public Health. 2018;15(1):e12763-1. doi: 10.2427/12763.

For peer review only - http://bmjopen.bmj.com/site/about/guidelines.xhtml

45. De Caterina R, Bruggenjurgen B, Darius H, Kohler S, Lucerna M, Pecen L, et al. Quality of life and patient satisfaction in patients with atrial fibrillation on stable vitamin K antagonist treatment or switched to a non-vitamin K antagonist oral anticoagulant during a 1-year follow-up: A PREFER in AF Registry substudy. Arch Cardiovasc Dis. 2018;111(2):74-84. Epub 2017/09/25. doi: 10.1016/j.acvd.2017.04.007. PubMed PMID: 28942115.
46. Kotecha D, Ahmed A, Calvert M, Lencioni M, Terwee CB, Lane DA. Patient-Reported Outcomes for Quality of Life Assessment in Atrial Fibrillation: A Systematic Review of Measurement Properties. PLoS One. 2016;11(11):e0165790. Epub 20161101. doi: 10.1371/journal.pone.0165790. PubMed PMID: 27802324; PubMed Central PMCID: PMCPMC5089715.
47. Reynolds MR, Ellis E, Zimetbaum P. Quality of life in atrial fibrillation: measurement tools and impact of interventions. J Cardiovasc Electrophysiol. 2008;19(7):762-8. Epub 20080205. doi: 10.1111/j.1540-8167.2007.01091.x. PubMed PMID: 18266667; PubMed Central PMCID: PMCPMC2574796.
48. Vibeke Høgh V, Delmar C, Riahi S, Kirsten Frederiksen K. Quality of Life - Exemplified through Patients Living with Atrial Fibrillation. J Nurs Care 2014; 3(2). doi: http://dx.doi.org/10.4172/2167-1168.1000150.
49. EQ-5D. EQ-5D-5L. About. Available from: https://euroqol.org/eq-5d-instruments/eq-5d-5l-about/ (Access date 13 January 2022) 2009.
50. Prins MH, Guillemin I, Gilet H, Gabriel S, Essers B, Raskob G, et al. Scoring and psychometric validation of the Perception of Anticoagulant Treatment Questionnaire (PACT-Q). Health Qual Life Outcomes. 2009;7:30. Epub 20090407. doi: 10.1186/1477-7525-7-30. PubMed PMID: 19348685; PubMed Central PMCID: PMCPMC2686675.
51. Prins MH, Marrel A, Carita P, Anderson D, Bousser MG, Crijns H, et al. Multinational development of a questionnaire assessing patient satisfaction with anticoagulant treatment: the 'Perception of Anticoagulant Treatment Questionnaire' (PACT-Q). Health Qual Life Outcomes. 2009;7:9. Epub 20090206. doi: 10.1186/1477-7525-7-9. PubMed PMID: 19196486; PubMed Central PMCID: PMCPMC2654430.
52. Lancaster GA, Dodd S, Williamson PR. Design and analysis of pilot studies: recommendations for good practice. J Eval Clin Pract. 2004;10(2):307-12. doi: 10.1111/j.2002.384.doc.x. PubMed PMID: 15189396.
53. Schulman S, Kearon C, Subcommittee on Control of Anticoagulation of the S, Standardization Committee of the International Society on T, Haemostasis. Definition of major bleeding in clinical investigations of antihemostatic medicinal products in non-surgical patients. J Thromb Haemost. 2005;3(4):692-4. Epub 2005/04/22. doi: 10.1111/j.1538-7836.2005.01204.x. PubMed PMID: 15842354.
[dataset] 54. Hutchings HA, Lanyon K, Holland G, Alikhan R, Jenkins R, Laing H, et al. Can we collect health-related quality of life information from anticoagulated atrial fibrillation participants who have recently experienced a bleed? An observational feasibility study in primary, and secondary care and through an online forum. Available from: https://zenodo.org/record/8147317. Zenodo2023.
55. Calvert MJ, Cruz Rivera S, Retzer A, Hughes SE, Campbell L, Molony-Oates B, et al. Patient reported outcome assessment must be inclusive and equitable. Nat Med. 2022;28(6):1120-4. doi: 10.1038/s41591-022-01781-8. PubMed PMID: 35513530.

For peer review only - http://bmjopen.bmj.com/site/about/guidelines.xhtml

FIGURES

Figure 1. Flow chart of the stages involved in the study.

Figure 2. Flow chart of the participants recruited to the study and subsequently analysed.

For peer review only

For peer review only - http://bmjopen.bmj.com/site/about/guidelines.xhtml

TABLES

For peer review only

For peer review only - http://bmjopen.bmj.com/site/about/guidelines.xhtml

Table 1. Demographics of the recruited participants

	Primary care (n=14)	AF forum (n=12)*
Ethnicity		
White	14(100%)	n/a
Sex (M:F)	3:11	n/a
CHA_2DS_2-VASc		
Mean (Min, Max)	3.64 (1, 6)	n/a
HAS-BLED		
0	1(7.1%)	n/a
1	5(35.7%)	
2	8(57.1%)	
Medication		
Apixaban	7(50.0%)	1(8.3%)
Dabigatran	0(0%)	1(8.3%)
Rivaroxaban	5(35.7%)	4(33.3%)
Warfarin	2(14.3%)	2(16.7%)
Xarelto	0(0%)	1(8.3%)
Missing	1(7.1%)	3(25.0%)
Co-morbidities/pre-existing disease		
Congestive heart failure	0(0%)	1(8.3%)
Hypertension	6(42.9%)	5(41.7%)
Hypotension	1(7.1%)	0(0%)
Ischaemic heart disease	0(0%)	1(8.3%)
Chronic Obstructive Pulmonary Disease	2(14.3%)	0(0%)
Diabetes related heart disease	1(7.1%)	1(8.3%)
Other	9(64.3%)	3(25.0%)
Days since bleed		
0-7	3(21.4%)	2(16.7%)
8-30	0(0%)	2(16.7)
31-60	1(7.1%)	4(33.3%)
60+	1(7.1%)	0(0%)
Unknown	9(64.3%)	4(33.3%)
Location of Bleed		
Internal	3(21.4%)	4(33.3%)
External	7(50%)	4((33.3%)
Unsure	1(7.1%)	1(8.3%)
Missing	3(21.4%)	3(25.0%)
Body location of external bleed		
Head	0(0%)	2(50.0%)
Neck	1(14.3%)	0(0%)
Forearm	2(28.6%)	0(0%)
Calf/shin	1(14.3%)	1(25.0%)
Feet, ankle, toes	1(14.3%)	0(0%)
Haemorrhoids	1(14.3%)	0(0%)
Missing	1(14.3%)	1(25.0%)
Body location of internal bleed		
Head	2(66.7%)	0 (0%)
Respiratory system	0(0%)	1(25.0%)

For peer review only

For peer review only - http://bmjopen.bmj.com/site/about/guidelines.xhtml

Reproductive system	0(0%)	1(25.0%)
Other	1(33.3%)	2(50.0%)
Frequency of minor bleeds		
1-2 times per week	4(28.6%)	1(8.3%)
1-2 times per month	2(14.3%)	4(33.3%)
1-2 times over 6 months	3(21.4%)	2(16.7%)
1-2 times a year	0(0%)	1(8.3%)
Almost never	3(21.4%)	2(16.7%)
Missing	2(14.3%)	2(16.7%)
Frequency of major bleeds		
1-2 per week	1(7.1%)	0(0%)
1-2 times a year	0(0%)	2(16.7%)
Almost never	10(71.4%)	7(58.3%)
Missing	3(21.4%)	3(25.0%)

- Demographic/symptom information was not collected on the AF forum as it could not be clinically verified.

For peer review only

Table 2. Health-related quality of life (HRQoL) scores at baseline and follow up split by primary care and AF forum patients measured using the EQ5D, AFEQT and PACT questionnaires.

a) EQ5D UK crosswalk scores

EQ-5D UK Crosswalk*						
Mean (Min, Max)	First Survey		Follow-up Survey		Total	
Primary Care	n=13	0.470 (-0.200, 1.000)	n=8	0.694 (0.393, 1.000)	n=21	0.555 (-0.200, 1.000)
Online Forum	n=12	0.809 (0.476, 1.000)	n=3	0.893 (0.679, 1.000)	n=15	0.826 (0.476, 1.000)
Total	n=25	0.633 (-0.200, 1.000)	n=11	0.748 (0.393, 1.000)	n=36	0.668 (-0.200, 1.000)

* The crosswalk has a score range of -0.594 – 1.000.

EQ-5D UK VAS						
Mean (Min, Max)	First Survey		Follow-up Survey		Total	
Primary Care	n=13	58.9 (22, 92)	n=8	60.1 (25, 98)	n=21	59.4 (22, 98)
Online Forum	n=12	70.6 (25, 92)	n=3	83.0 (70, 90)	n=15	73.1 (25, 92)
Total	n=25	64.5 (22, 92)	n=11	66.4 (25, 98)	n=36	65.1 (22, 98)

b) AFEQT Scores

AFEQT		
Mean (Min, Max)	First Survey	Follow-up Survey
Global Score		
Primary Care	64.8 (18.5, 98.1)	63.3 (37.0, 91.7)
AF Forum	65.7 (25.9, 94.4)	77.2 (50.5, 93.5)
Symptom Domain Score		
Primary Care	76.4 (33.3, 100)	74.0 (37.5, 95.8)
AF Forum	78.0 (37.5, 100)	81.9 (58.3, 95.8)
Daily Activities Domain Score		
Primary Care	50.0 (14.6, 100)	59.8 (8.3, 95.8)
AF Forum	58.7 (10.4, 91.7)	76.4 (47.9, 95.8)
Treatment Concern Domain Score		
Primary Care	76.9 (13.9, 100)	59.1 (11.1, 83.3)
AF Forum	66.9 (33.3, 91.4)	75.0 (50.0, 91.7)

AFEQT Satisfaction Questions		
Mean (Min, Max)	First Survey	Follow-up Survey
Q19. How well your current treatment controls your atrial fibrillation?		
Primary Care	75.0 (50.0, 100)	62.5 (16.7, 100)
AF Forum	65.3 (0.0, 83.3)	61.1 (50.0, 66.7)
Q20. The extent to which treatment has relieved your symptoms of atrial fibrillation?		

For peer review only - http://bmjopen.bmj.com/site/about/guidelines.xhtml

Primary Care	73.6 (50.0, 100)	62.5 (16.7, 83.3)
AF Forum	66.7 (0.0, 100)	61.1 (50.0, 66.7)

c) PACT-Q Part 2 Scores

PACT-Q Scores		
Mean (Min, Max)	First Survey	Follow-up Survey
Convenience score		
Primary Care	87.0 (59.6, 100)	86.8 (69.2, 100)
AF Forum	86.5 (65.4, 98.1)	86.5 (76.9, 98.1)
Satisfaction score		
Primary Care	65.4 (39.3, 96.4)	61.6 (50.0, 78.6)
AF Forum	67.5 (39.3, 89.3)	76.2 (64.3, 92.9)

For peer review only

For peer review only - http://bmjopen.bmj.com/site/about/guidelines.xhtml

Figure 1. Flow chart of the stages involved in the study

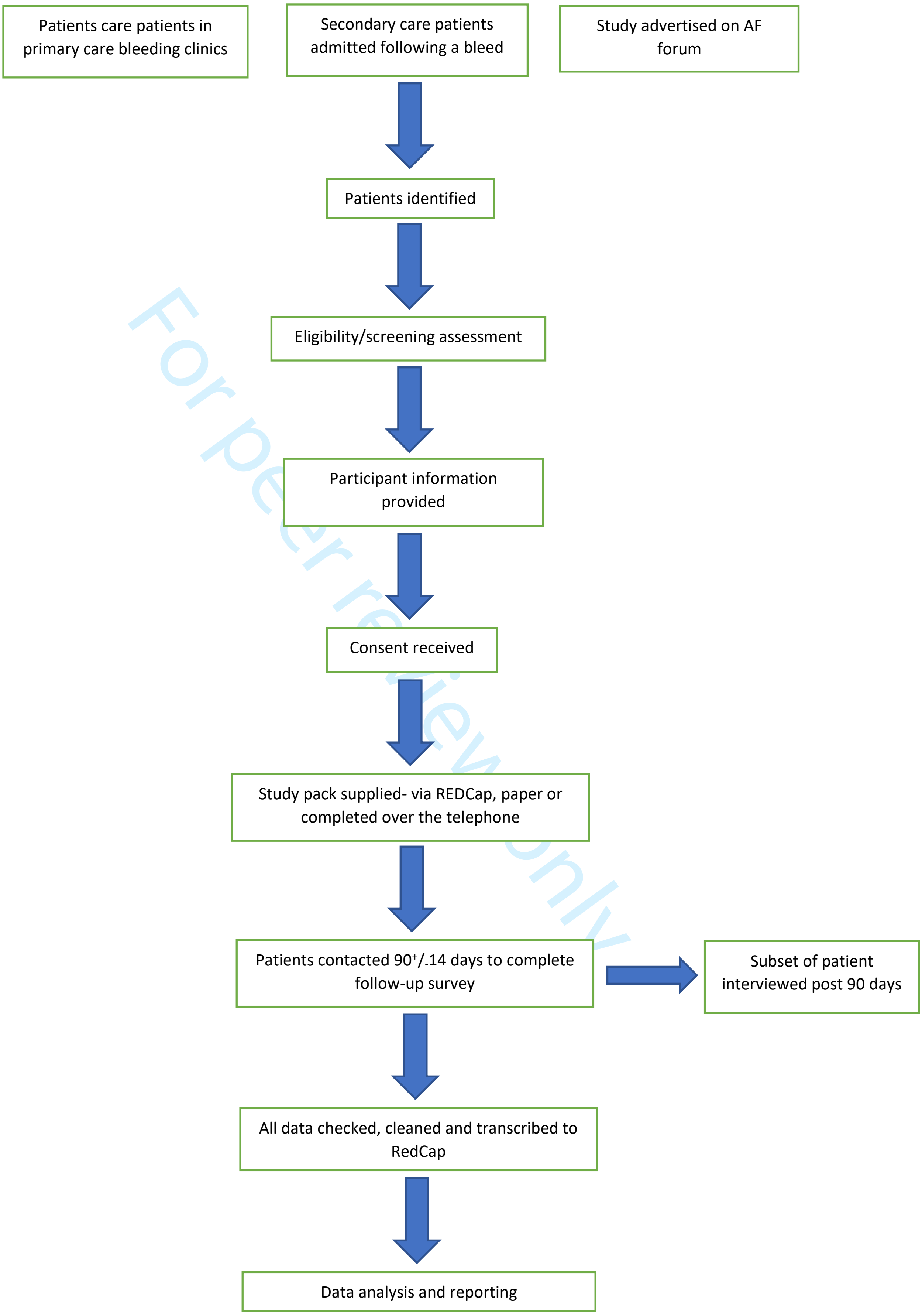

For peer review only

Figure 2. Flow chart of the participants recruited to the study and subsequently analysed

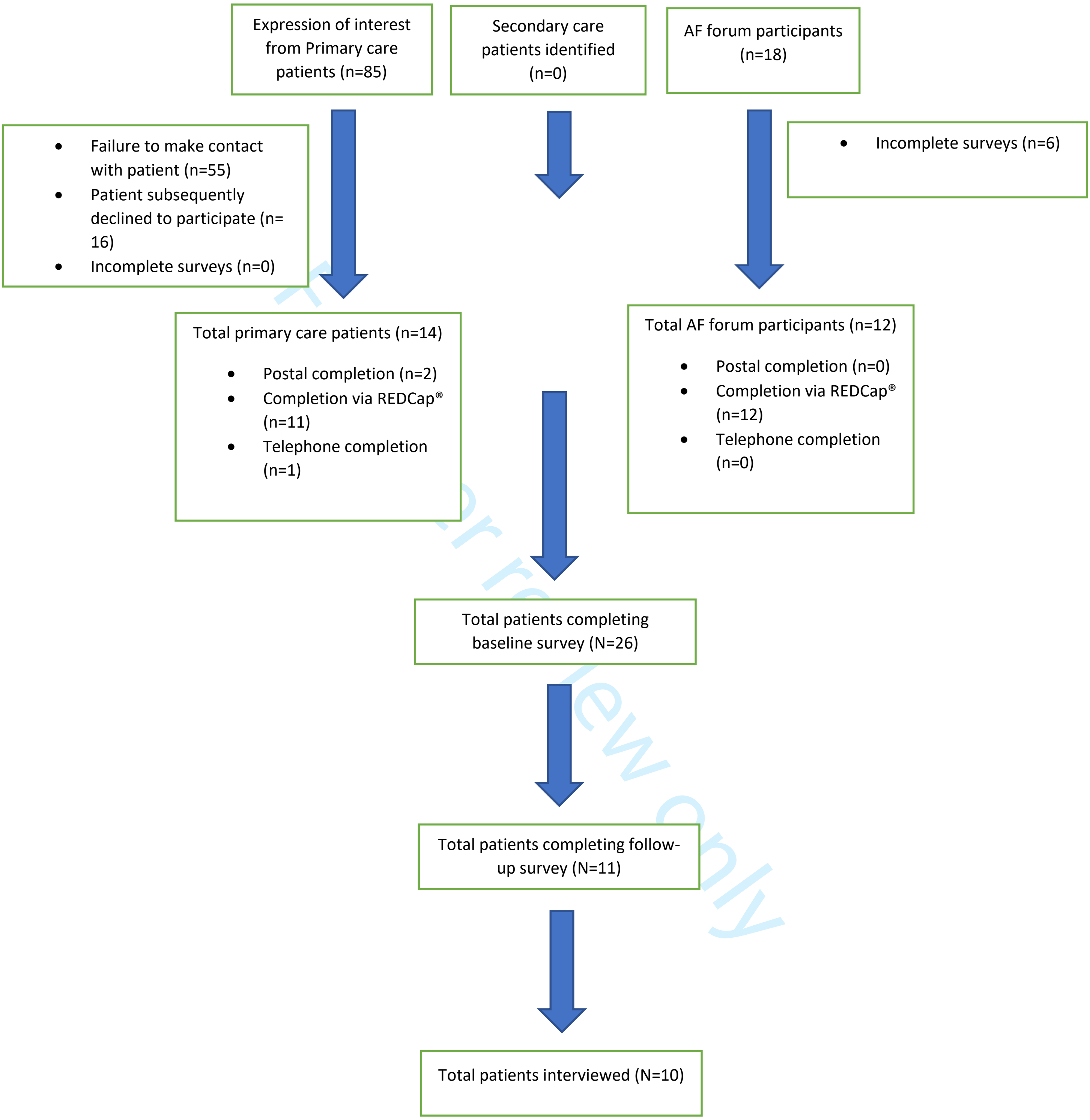

STROBE Statement—Checklist of items that should be included in reports of ***cross-sectional studies***

	Item No	Recommendation	Page No
**Title and abstract**	1	(*a*) Indicate the study's design with a commonly used term in the title or the abstract	1
		(*b*) Provide in the abstract an informative and balanced summary of what was done and what was found	2-3
**Introduction**			
Background/rationale	2	Explain the scientific background and rationale for the investigation being reported	5
Objectives	3	State specific objectives, including any prespecified hypotheses	5
**Methods**			
Study design	4	Present key elements of study design early in the paper	8
Setting	5	Describe the setting, locations, and relevant dates, including periods of recruitment, exposure, follow-up, and data collection	7
Participants	6	(*a*) Give the eligibility criteria, and the sources and methods of selection of participants	6
Variables	7	Clearly define all outcomes, exposures, predictors, potential confounders, and effect modifiers. Give diagnostic criteria, if applicable	8
Data sources/ measurement	8*	For each variable of interest, give sources of data and details of methods of assessment (measurement). Describe comparability of assessment methods if there is more than one group	*8*
Bias	9	Describe any efforts to address potential sources of bias	7
Study size	10	Explain how the study size was arrived at	8
Quantitative variables	11	Explain how quantitative variables were handled in the analyses. If applicable, describe which groupings were chosen and why	8
Statistical methods	12	(*a*) Describe all statistical methods, including those used to control for confounding	8
		(*b*) Describe any methods used to examine subgroups and interactions	N/A
		(*c*) Explain how missing data were addressed	8
		(*d*) If applicable, describe analytical methods taking account of sampling strategy	N/A
		(*e*) Describe any sensitivity analyses	N/A
**Results**			
Participants	13*	(a) Report numbers of individuals at each stage of study—eg numbers potentially eligible, examined for eligibility, confirmed eligible, included in the study, completing follow-up, and analysed	10-11
		(b) Give reasons for non-participation at each stage	10
		(c) Consider use of a flow diagram	20
Descriptive data	14*	(a) Give characteristics of study participants (eg demographic, clinical, social) and information on exposures and potential confounders	22-23
		(b) Indicate number of participants with missing data for each variable of interest	22-23
Outcome data	15*	Report numbers of outcome events or summary measures	22-23

For peer review only

For peer review only - http://bmjopen.bmj.com/site/about/guidelines.xhtml

Main results	16	(*a*) Give unadjusted estimates and, if applicable, confounder-adjusted estimates and their precision (eg, 95% confidence interval). Make clear which confounders were adjusted for and why they were included	N/A
		(*b*) Report category boundaries when continuous variables were categorized	24-25
		(*c*) If relevant, consider translating estimates of relative risk into absolute risk for a meaningful time period	N/A
Other analyses	17	Report other analyses done—eg analyses of subgroups and interactions, and sensitivity analyses	N/A
**Discussion**			
Key results	18	Summarise key results with reference to study objectives	12
Limitations	19	Discuss limitations of the study, taking into account sources of potential bias or imprecision. Discuss both direction and magnitude of any potential bias	12-13
Interpretation	20	Give a cautious overall interpretation of results considering objectives, limitations, multiplicity of analyses, results from similar studies, and other relevant evidence	12-13
Generalisability	21	Discuss the generalisability (external validity) of the study results	12-13
**Other information**			
Funding	22	Give the source of funding and the role of the funders for the present study and, if applicable, for the original study on which the present article is based	14

*Give information separately for exposed and unexposed groups.

Note: An Explanation and Elaboration article discusses each checklist item and gives methodological background and published examples of transparent reporting. The STROBE checklist is best used in conjunction with this article (freely available on the Web sites of PLoS Medicine at http://www.plosmedicine.org/, Annals of Internal Medicine at http://www.annals.org/, and Epidemiology at http://www.epidem.com/). Information on the STROBE Initiative is available at www.strobe-statement.org.

For peer review only
